# Mammalian Browsers Disrupt Eco‐Evolutionary Dynamics in a Forest Tree Restoration Planting

**DOI:** 10.1111/eva.70099

**Published:** 2025-05-07

**Authors:** João Costa e Silva, Brad M. Potts, Peter A. Harrison

**Affiliations:** ^1^ Centro de Estudos Florestais, Instituto Superior de Agronomia Universidade de Lisboa Lisboa Portugal; ^2^ School of Natural Sciences University of Tasmania Hobart Tasmania Australia; ^3^ ARC Training Centre for Forest Value University of Tasmania Hobart Tasmania Australia; ^4^ EcoAdapt Consulting Pty Ltd Old Beach Tasmania Australia

**Keywords:** assisted migration, climate adaptation and modelling, differentiation of tree species and provenances, family variance, herbivore‐plant interactions, tree survival and height growth

## Abstract

Native and restored forests are increasingly impacted by pests and diseases, including large herbivores. While community‐ and species‐level impacts of these tree enemies are often well‐documented, there is little understanding of their influence on finer‐scale eco‐evolutionary processes. We here study the influence of large‐mammal herbivory on the survival and height growth of trees in a mixed species restoration planting of the Australian forest trees, *Eucalyptus ovata* and *E. pauciflora* , in Tasmania, Australia. Common‐garden field trials mixing the two species were compared in adjacent unbrowsed (fenced) and browsed (unfenced) plantings. The browsed planting was exposed to mammal browsing by native marsupials, as well as feral introduced European fallow deer ( *Dama dama* ). Each tree species was represented by open‐pollinated families from 22 paired geographic areas, allowing the assessment of the effects of browsing on the species and population differences, as well as on family variation within each species. In the browsed planting, a marked reduction in species and population differences, as well as in family variance, was observed for both height growth and survival. The pattern of height growth and survival of the populations of both species also differed between browsing regimes, with significant changes of climate relationships involving both focal tree attributes detected. Our results argue for a major disruption of the eco‐evolutionary dynamics of restored forests in the presence of browsing by large mammalian herbivores, at the observed period of the tree life cycle. Importantly for forest restoration and conservation in the face of global change, our results challenge the choice of tree populations for translocation based solely on predicted or observed relationships of their home‐site climate with current and predicted future climates of the restoration sites, while emphasising the need for genetic diversity to provide future resilience of restored forests to both biotic and abiotic stresses.

## 1 Introduction

Predicting the outcomes of ecological restoration is notoriously difficult (Grman et al. 2013). Species and provenance choices are important early decisions (Erickson and Halford 2020; Thomas et al. 2014) but challenging in the face of global climate change, anthropogenic habitat modification and novel biotic interactions (Heenan et al. 2023; Moreno‐Mateos et al. 2020). While using local seed sources was once the default strategy, seed sourcing strategies have now changed to accommodate these new eco‐evolutionary risks (Jordan et al. 2024; Prober et al. 2019). Such risks refer to the ecological and evolutionary interactions that occur on a contemporary time scale (Hendry 2017). In the case of ecological restoration, provenancing strategies now advocate mixing seed from local and non‐local sources to enhance the adaptive capacity and resilience of plantings (Harrison, Breed, et al. 2021). These strategies aim to increase genetic diversity and adaptive variation in general—for example, composite or admixture provenancing (Bucharova et al. 2019)—or have a more specific target, such as increasing adaptation to the future climate of the restoration site (Aitken and Bemmels 2016; Prober et al. 2015; Stanturf et al. 2024). Climate‐adjusted provenancing, for example, aims to enrich local provenances with seed from provenances with home‐site climates aligned with the direction of future climate change predicted for the restoration site (Prober et al. 2015). This strategy requires understanding of within‐species variability in climate adaptation, as well as forecasts of the future climate of the planting site (Harrison et al. 2017). These contemporary provenancing strategies are particularly relevant to restoration or other conservation and commercial plantings involving long‐lived sedentary organisms such as forest trees, where planted individuals are expected to be exposed to a wide spectrum of novel abiotic and biotic risks over their lifetime (Alfaro et al. 2014; Hoffmann et al. 2021; Sgro et al. 2011).

While climate change vulnerability has been a focus of much of the forest restoration and genetic conservation literature (Ramírez‐Valiente et al. 2022; Sgro et al. 2011; Vitt et al. 2022), biotic stresses are also important (Alfaro et al. 2014; Bryant et al. 2024; Champagne, Royo, et al. 2021; Jordan et al. 2024; Stanturf 2015; Xu et al. 2023). When their effects on tree fitness are genetically based, tree enemies may shape the structure, function and long‐term resilience of forest stands (Cope et al. 2021). The risks posed by native enemies are often dependent on climate, and thus confounded with general climate adaptation (Hamann et al. 2021; Pinkard et al. 2010; Sturrock et al. 2011). However, with human‐facilitated translocations and global change, these biotic stresses increasingly involve exotic enemies, which have not co‐evolved with the local tree species (Freeman et al. 2019; Garbelotto and Pautasso 2012; Ghelardini et al. 2022; Nahrung and Carnegie 2020; Panzavolta et al. 2021). Mammal browsers are a major threat to the success of forest regeneration and ecological restoration in many countries (Champagne, Raymond, et al. 2021; Nilar et al. 2019; Reid et al. 2023; Zerga 2015). In Australia, the browsing risk is often from both native marsupials and introduced mammals (Gordon et al. 2019; Nilar et al. 2019; Reid et al. 2023). This is certainly the case on the southern island of Tasmania, where the necessity of tree protection from mammalian browsing (e.g., with exclosures) is well‐documented for eucalypt forest and woodland restoration on converted grazing lands (Bailey et al. 2014; Bailey, Harrison, Hanusch, et al. 2021; Davidson, Bailey, Burgess, and Potts 2021; Harrison, Davidson, et al. 2021).

For the Tasmanian restoration sites, climate modelling has been undertaken to predict the native tree species (Harrison 2021) and their provenances (Harrison et al. 2017) most suited to the contemporary and forecasted future climates. Multi‐site field trials have been established to test these predictions (Bailey, Harrison, Davidson, et al. 2021), with one experiment established to specifically understand the extent to which these climate‐based predictions are likely to be modified by large (> 2 kg, Pringle et al. 2023) mammalian browsers. These browsers include native marsupials (e.g., wallabies and possums), which are a common threat to the Tasmanian restoration plantings (Bailey, Harrison, Davidson, et al. 2021; Davidson, Bailey, Burgess, and Potts 2021). Heavy damage to young trees in these plantings can also be caused by the feral European fallow deer ( *Dama dama* ; Bailey et al. 2014; Bailey, Harrison, Davidson, et al. 2021). Importantly, species and provenance differences in eucalypt susceptibility to deer damage in field trials have been reported (Bailey et al. 2014; Bailey, Harrison, Davidson, et al. 2021; Camarretta, Harrison, Bailey, et al. 2020), highlighting their potential to affect eco‐evolutionary processes. Understanding the potential of large mammalian browsers to affect eco‐evolutionary trajectories of restored forests is important in the broader context, given their impact on ecosystems world‐wide (Lacher Jr et al. 2019; Leal et al. 2022; Pringle et al. 2023) and the relevance of biotic interactions in general in determining outcomes of ecological restoration (Hoffmann et al. 2021; Jordan et al. 2024; Sgro et al. 2011) and assisted translocations (Champagne, Raymond, et al. 2021).

*Eucalyptus ovata* and *E. pauciflora* are two of the main evergreen tree species used in restoration plantings in the drier, central regions of Tasmania and included in embedded field trials (Bailey, Harrison, Hanusch, et al. 2021). We here use measurements of 3‐year height growth and 8‐year tree survival to compare adjacent unbrowsed and browsed field trials of open‐pollinated families of both species sourced from multiple native provenances in Tasmania. We aim to understand how exposure to biotic enemies, specifically large mammalian herbivores, is likely to modify species and provenance choices of the key restoration eucalypts and affect the eco‐evolutionary dynamics of restored forests. Given the possibility of growth‐defence trade‐offs (Cope et al. 2021), we specifically hypothesise that mammalian browsing will disrupt signals of local climate adaptation at the species, provenance (hereafter denoted as ‘population’) and family levels, and that different eucalypt seed sources will be favoured depending upon the browsing regime.

## 2 Methods

### 2.1 Field Trials and Measurements

Two common‐garden field trials, each comprising inter‐mixed plantings of *E. ovata* and *E. pauciflora* families, were established in August 2014 in fenced and unfenced reforestation plantings on an ex‐pasture site adjacent to remnant native forest at ‘Connorville’ near Cressy, northern Tasmania, Australia (41.828° S, 147.138° E, altitude of 185 m; for further details, see Bailey, Harrison, Davidson, et al. 2021; Prober et al. 2022). *E. ovata* and *E. pauciflora* would have been a component of the woodland/forest originally cleared for pasture establishment, with remnant trees fragmented throughout the agricultural matrix (Fensham 1989). Each species was represented by a reasonably large number of common families across the two trials (i.e., the number of common families was 147 for *E. ovata* and 150 for *E. pauciflora* ; Table S1), and as the trials were in close proximity (< 500 m), their climate and soil conditions were considered to be equivalent. Accordingly, the two mixed species trials were treated as only differing in their ‘browsing regime’ defined in terms of protection from (unbrowsed) or exposure to (browsed) browsing by large (> 2 kg) mammals, the most notable of which was the exotic fallow deer, which can cause heavy damage to young trees (Bailey et al. 2014; Bailey, Harrison, Davidson, et al. 2021). These deer were introduced into Tasmania in the 1930's, but their numbers have expanded 40‐fold and their core distribution increased 3‐fold since 1985 (Botterill‐James et al. 2024; Cunningham et al. 2022). Their core distribution now encompasses the rural landscapes, which have been a focus of ecological restoration activity for over a decade (Davidson, Bailey, and Burgess 2021). The planted eucalypts were also subject to browsing by native marsupials, specifically the common brushtail possum ( *Trichosurus vulpecula* ), the Tasmanian pademelon ( *Thylogale billardierii* ) and the Bennetts wallaby (*Notamacropus rufogriseus*; Bailey et al. 2014; Bailey, Harrison, Davidson, et al. 2021). These marsupials shelter in native forest or remnant trees and browse on the introduced grasses in the adjacent grazing lands (Wiggins and Bowman 2011).

**FIGURE 1:**
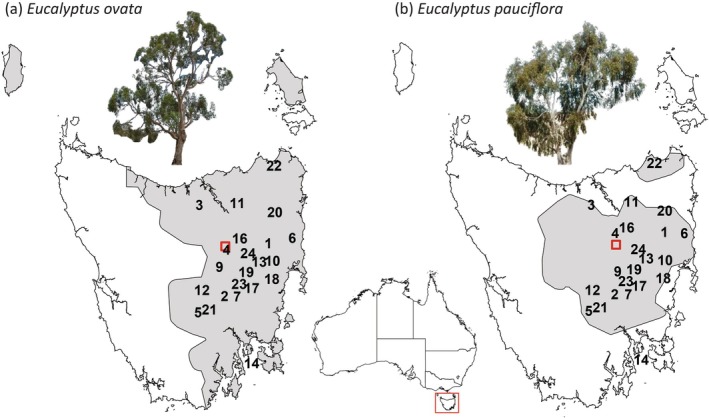
Maps of the Tasmanian geographic range of (a) *Eucalyptus ovata* and (b) *E. pauciflora* . The natural range, distribution of the sampled 22 paired geographic areas (black points; see Table S2) and the common‐garden trial site at Connorville (red box) are shown. The region mapped is indicated in the insert of Australia. (Images: R. Wiltshire and P.A. Harrison).

For both eucalypt species, the planted families were derived from open‐pollinated seed, obtained from well‐spaced (> 100 m) mother trees sampled in native populations across their eastern geographic range in Tasmania. The populations of both species were sampled in pairs from each of 22 specific geographic areas, which were chosen to represent regions where the species' natural distributions overlapped (Figure 1; Table S2). While the species sometimes grow in close proximity, they generally occupy different local habitats, with *E. ovata* occurring on seasonally waterlogged soils and *E. pauciflora* on the better drained soils (Williams and Potts 1996). However, the sampling strategy ensured that the macro‐climatic gradients experienced by the populations from the two species in each pair were effectively the same (Prober et al. 2022), and thus we treated a given pair of populations as a single geographic area common to both species.

Within each field trial, the families of each species were arranged randomly and independently of their population of origin, using an experimental complete block design comprising eight blocks. Each block was arranged as a rectangular grid with an inter‐tree spacing of 3 m (between rows) by 2.5 m (within rows), and each family was represented as a single‐tree plot. The experimental designs of each species were then superimposed spatially, with each alternate position along a row planted with a different species, and rows offset so that the four nearest neighbours of a focal tree were trees of the other species (Bailey, Harrison, Davidson, et al. 2021; an example of this field layout is given in Figure S1). There was a moderate level of imbalance in the family representation and the number of individuals per family planted due to seedling availability and replacements; per species and browsing regime, the total number of deployed families varied from 147 to 157, and the total number of trees planted ranged from 1255 to 1273 (Table S1). The number of families represented per population of each species ranged between 5 and 13.

The assessment of height growth was undertaken at age 3 years. Growth performance was assessed by using maximum tree height (m), measured with height poles. At this stage, most surviving trees in the browsed planting exhibited some signs of deer damage (i.e., 61% in *E. ovata* , and 83% in *E. pauciflora* ), whereas there was minimal damage on trees in the unbrowsed planting (< 0.2% of the trees exhibited such damage). At age 8 years from planting, tree survival was scored as a binary outcome whereby a tree was classified as dead (scored as 0) when no live plant tissue was evident above ground or the plant was in very poor vegetative health, otherwise it was classified as alive (scored as 1).

### 2.2 Data Analysis

#### 2.2.1 Analyses of Tree Survival and Height Growth Using Mixed‐Effects Models

For each species and browsing regime, our data had a hierarchical structure with three levels: (level‐1) individual trees clustered within (level‐2) families, which were clustered within (level‐3) geographic areas. Although it may have analogies with the ‘population’ or ‘provenance’ concepts commonly applied in forestry (Eldridge et al. 1993) and tree restoration (Bailey, Harrison, Davidson, et al. 2021; Harrison, Breed, et al. 2021) field trials, the terminology ‘geographic area’ was used in our modelling to refer to the paired sampling of each species (i.e., a single geographic area was represented by a pair of populations, one from *E. ovata* and the other from *E. pauciflora* ). Following the approach described by McNeish and Wentzel (2017) for modelling cross‐sectional clustered data with three hierarchical levels, the analyses of tree survival and height growth used a two‐level, mixed‐effects model with fixed‐effect dummy variables for geographic areas and random intercepts for family effects. Thus, geographic area was modelled as a fixed‐effect term (since we were interested in testing the difference among the specifically chosen regions within the eastern geographic range in Tasmania, where the species' natural distributions overlapped) to accommodate the highest level of the data hierarchy, whereas families within geographic areas were modelled as random effects to account for the second level of clustering.

In Methods S1, we provide the details on the definition and estimation of the two‐level, mixed‐effects models applied to analyse the data combined across the two studied field trials (i.e., across species and browsing regimes), where a generalized linear mixed model (GLMM) using the probit link function was fitted to the binary variable (tree survival) and a linear mixed model (LMM) was fitted to the continuous variable (height growth). The fixed‐effect parameters in these models included blocks within species and browsing regime, and the effects associated with the model terms of species, browsing regime, geographic area, and their two‐ and three‐way interactions. Under this model specification, the analyses of survival and height modelled a variance–covariance matrix of the random effects that accounted for heterogeneity of family variances among species and browsing regimes. Within a species, a final fitted model comprised also a covariance between browsing regimes for a given family, but only when the family variances had been previously found to be statistically significant (*p* < 0.05) in both browsing regimes simultaneously (see Methods S1 for details on the statistical testing of the family variance parameter in separate analyses for each combination of species and browsing regime). In addition, in the analysis of height, we modelled separate residual variances for a given combination of species and browsing regime, hence allowing for a heterogeneous residual variance structure.

In the across‐trial analyses described above, estimation of (co)variance parameters under the GLMM fitted to survival relied on the residual solution pseudo‐likelihood (RSPL) method (Wolfinger and O'Connell 1993) and restricted maximum likelihood (REML) estimation (Patterson and Thompson 1971) was used in the LMM fitted to height. However, for either response variable, a preliminary estimation of family variances was undertaken in separate analyses for each combination of species and browsing regime under a reduced two‐level, mixed‐effects model with blocks and geographic areas as fixed‐effect parameters, and random intercepts for families within geographic areas. Then, the estimated (RSPL or REML) family variances from these separate analyses were used as initial parameter values in the estimation of the final GLMM and LMM models defined to fit the survival and height data, respectively, combined across species and browsing regimes.

In GLMMs for binary outcomes, a linearisation‐based approach such as RSPL may offer a computationally advantageous alternative to maximum likelihood (ML) estimation, especially with a moderately large number of cluster entities and a complex model specification involving multiple (eventually correlated) random parameters (Hedeker and Gibbons 2006; Kim et al. 2013), as in the GLMM fitted to survival data combined across trials (Methods S1). However, reported analyses of binary responses by three‐level GLMMs modelling only two variance parameters—that is, random intercepts at the levels 3 and 2 of the data hierarchy—indicated that linearisation‐based methods may particularly underestimate level‐2 variances, when compared to nearly unbiased ML estimates obtained from Gaussian quadrature (Pinheiro and Chao 2006; Rodriguez and Goldman 2001; see Methods S1, for further information and references). Accordingly, in separate analyses of survival for each combination of species and browsing regime, we also pursued ML parameter estimation by using an adaptive Gaussian quadrature (AGQ) algorithm (Pinheiro and Chao 2006) under a three‐level probit GLMM with random intercepts for geographic areas and families within geographic areas, and blocks as fixed effects. The intention of these analyses was to use the ML family variance estimates for two purposes: (i) as a benchmark for comparison with the RSPL family variances estimated for survival under the reduced two‐level probit GLMM defined separately for each combination of species and browsing regime (as described in the previous paragraph); and (ii) to undertake a one‐sided likelihood‐ratio test (Self and Liang 1987) to assess whether a family variance parameter for survival was significantly greater than zero, as the AGQ approximates the likelihood function thus enabling the deviance to be calculated (which is not feasible with RSPL; Hedeker and Gibbons 2006). In addition, for comparison with the ML family variance estimates, we also conducted Bayesian estimation of model parameters in separate analyses of survival for each combination of species and browsing regime under the three‐level GLMM definition used for ML estimation (see Methods S1 for methodological details).

In the mixed‐effects model defined to fit the survival or height data combined across trials, estimates of the fixed‐effect parameters were obtained by generalized least squares, and the Kenward and Roger (2009) methods were applied to approximate the denominator degrees of freedom in (partial) *F*‐tests pursued for statistical inference about the fixed‐effect model terms. For each species and browsing regime, as well as for each geographic area within a given species and browsing regime, least‐squares means and their 95% confidence limits were estimated for survival and height. In particular, for survival, the least‐squares means and their 95% confidence limits were estimated on the linked (probit) scale, and then converted to the expected probability scale via the inverse link transformation. The procedures GLIMMIX and MCMC implemented in the SAS 9.4 software (SAS 2017) were used in the frequentist and Bayesian data analyses, respectively.

#### 2.2.2 Modelling the Relationship of Height and Survival With Climate

##### 2.2.2.1 Climate Data and Climate Variable Choice

**FIGURE 2:**
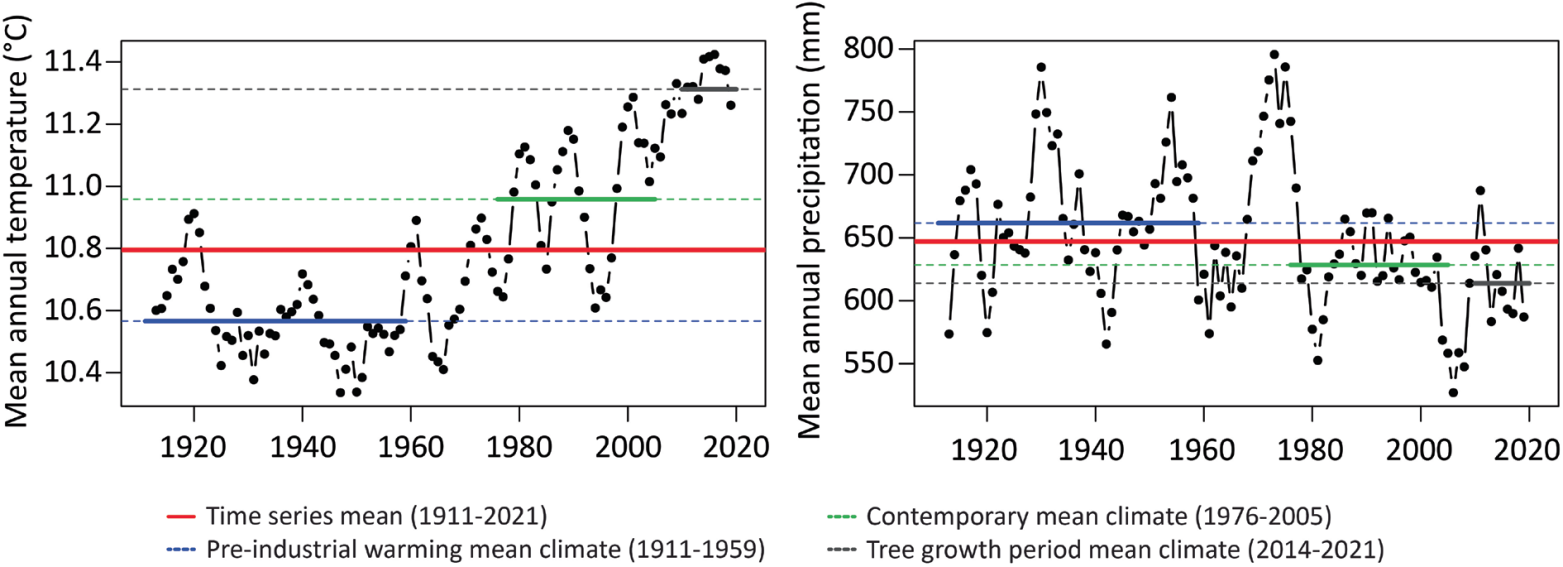
Long‐term climate patterns for the Connorville site where mixed species field trials of *Eucalyptus ovata* and *E. pauciflora* were established under two browsing regimes. The graphs show the 5‐year moving average mean annual temperature (TANN, °C) and annual precipitation (RANN, mm). The red line represents the overall average for the site (1911–2021), the blue line represents the historical climate average prior to the detectable signature of climate change in the southern hemisphere (Abram et al. 2016; pre‐industrial warming, 1911–1959), the green line corresponds to the climate average often used to represent the contemporary climate (1976–2005) and the black line corresponds to the climate average for the growing period from planting (2014–2021).

For studying how the browsing regime affected the relationship of height growth or tree survival with climate, we used climatic surfaces to calculate the standard 19 BIOCLIM variables (11 temperature and 8 rainfall variables; Methods S2) from daily precipitation and minimum and maximum temperatures from the 1 January 1911 until the 31 December 2021, downloaded from the Australian Bureau of Meteorology (http://www.bom.gov.au/climate/maps/, accessed on the 19 March 2024) and post‐analysed using the AUSClim‐R package (PA Harrison, unpublished) as detailed by Pfeilsticker et al. (2022). Briefly, daily data were extracted for each geographic area and used to calculate yearly BIOCLIM variables, which were then aggregated to the common climate period that represents the contemporary climate (1976–2005, e.g., ANUCLIM, version 6.1; Xu and Hutchinson 2013). The same bioclimatic data was also obtained for the Connorville common‐garden site, and climate averages were additionally calculated for: (i) the period prior to the detectable signature of climate change in the southern hemisphere (1911–1959 termed ‘pre‐industrial warming’, Abram et al. 2016); and (ii) the growing period climate experienced by the plants from planting (2014–2021). The growing period climate at the Connorville site was on average hotter and drier than its pre‐industrial warming and contemporary climates (Figure 2).

Using the contemporary climate averages, we summarised the climate variation among the studied 22 geographic areas with a principal component (PC) analysis of the correlation matrix among the standardised 19 BIOCLIM variables (see Methods S2). Following the approach described by Costa e Silva et al. (2018), we then identified the climatic variables that had the Pearson correlation coefficient (*r*) with the highest absolute magnitude with each of the first three PC axes (Methods S2). Accordingly, three temperature variables—maximum temperature of the warmest period (TMXWP, with period being a week in the present case; hereafter referred to as ‘maximum temperature’), mean temperature of the coldest quarter (TCLQ; hereafter referred to as ‘winter temperature’) and isothermality (TISO)—and one rainfall variable—precipitation of the warmest quarter (RWMQ; hereafter referred to as ‘summer rainfall’) ‐ were selected to describe the variation in contemporary climate among the geographic areas studied. An increase in TMXWP and a decrease in RWMQ reflected the aridity gradient described by PC1 (44%); a decrease in TCLQ reflected the cold stress gradient described by PC2 (27%); and an increase in TISO reflected the increase in diurnal temperature range relative to the seasonal range, indicative of a more continental‐type climate, as described by the PC3 (16%). These four climate variables, as well as the more traditional variables of annual precipitation (RANN) and annual mean temperature (TANN), were the focus of our analysis of the relationship of climate with height growth or tree survival. The growing period climate at the Connorville common‐garden site was on average hotter than the home‐site contemporary climate at the geographic areas studied (e.g., TMXWP, TCLQ and TANN; Methods S3).

##### 2.2.2.2 Height‐Climate Relationship

For each species and browsing regime, we modelled the relationship between the least‐squares mean for height growth of a geographic area and each focal climate variable. This was pursued by using a Gaussian generalized additive model (GAM) with an identity link function and a penalised thin‐plate smoother, fitted with the ‘gam’ function of the *mgcv* package in R (Wood 2017; Methods S4). In this analysis, the response observations were weighted by the inverse of the squared values of their estimated standard errors and normalised by dividing the weights by their mean following Wood (2017). Observations with high influence or leverage were iteratively removed to ensure that final models adequately met the assumptions of residual normality and homoscedasticity (detailed in Methods S4). Fitted curves and associated 95% confidence intervals (CIs) from the final models were standardised to a unit variance for plotting to aid comparisons across species and browsing regimes.

To test if the thin‐plate smoother of the relationship of height growth with each climate variable differed between species and browsing regime, an ordered‐factor smooth interaction GAM was used (detailed in Methods S4). In this context, the models we have fitted included geographic area (22 levels) and an ordered factor with four levels comprising unique combinations of species and browsing regime. Four contrasts of interest were constructed in the fitted models, namely comparing the smooth relationship between: (i) the browsing regimes for *E. ovata* ; (ii) the browsing regimes for *E. pauciflora* ; (iii) the two species in the unbrowsed planting; and (iv) the two species in the browsed planting. To evaluate the approximate statistical significance of these contrasts, a Wald *F*‐test was pursued using the ‘summary.gam’ function of the *mgcv* package in R.

##### 2.2.2.3 Survival‐Climate Relationship

The relationship of tree survival with each focal climate variable was similarly evaluated separately for each species and browsing regime (Methods S5). In this case, the values of the response variable were the estimated least‐squares means of the expected probability of survival for each geographic area, obtained from the GLMM (see above). In this context, by lying in a continuous scale within the interval (0, 1), the response variable was assumed to follow a standard beta distribution, which can be parameterised as a function of a location parameter μ (i.e., mean response) and a scaling parameter φ (i.e., ‘precision’ or the inverse of dispersion; Smithson and Verkuilen 2006). We fitted φ as a nuisance parameter (e.g., Ferrari and Cribari‐Neto 2004), and the probit link function was used for relating the linear predictor to the conditional mean (μ). The linear predictor was defined with the same model terms as previously used for the height‐climate relationship (except for an explicit residual term), and the response variable was weighted as previously described.

Estimates of the GAM parameters were obtained by maximum likelihood estimation. The final fitted models excluded observations that were detected to have substantial leverage and influence (based on the diagnostics and graphic examination described in Methods S4). Statistical significance of the survival‐climate relationships on the probit scale was approximated by undertaking a Wald Chi‐square test, using the ‘summary.gam’ function of the *mgcv* package in R. Plots of fitted curves and associated 95% CIs based on the final models were obtained on the probability scale (i.e., via the inverse probit link function), and standardised to a unit variance. To test if the smoother of the survival‐climate relationship for each climate variable differed between species and browsing regime, an ordered‐factor smooth interaction GAM was pursued similarly to what was described above for height. Subsequently, the approximate statistical significance of each of the four contrasts of interest was assessed using a Wald Chi‐square test.

## 3 Results

### 3.1 Species Level

**FIGURE 3:**
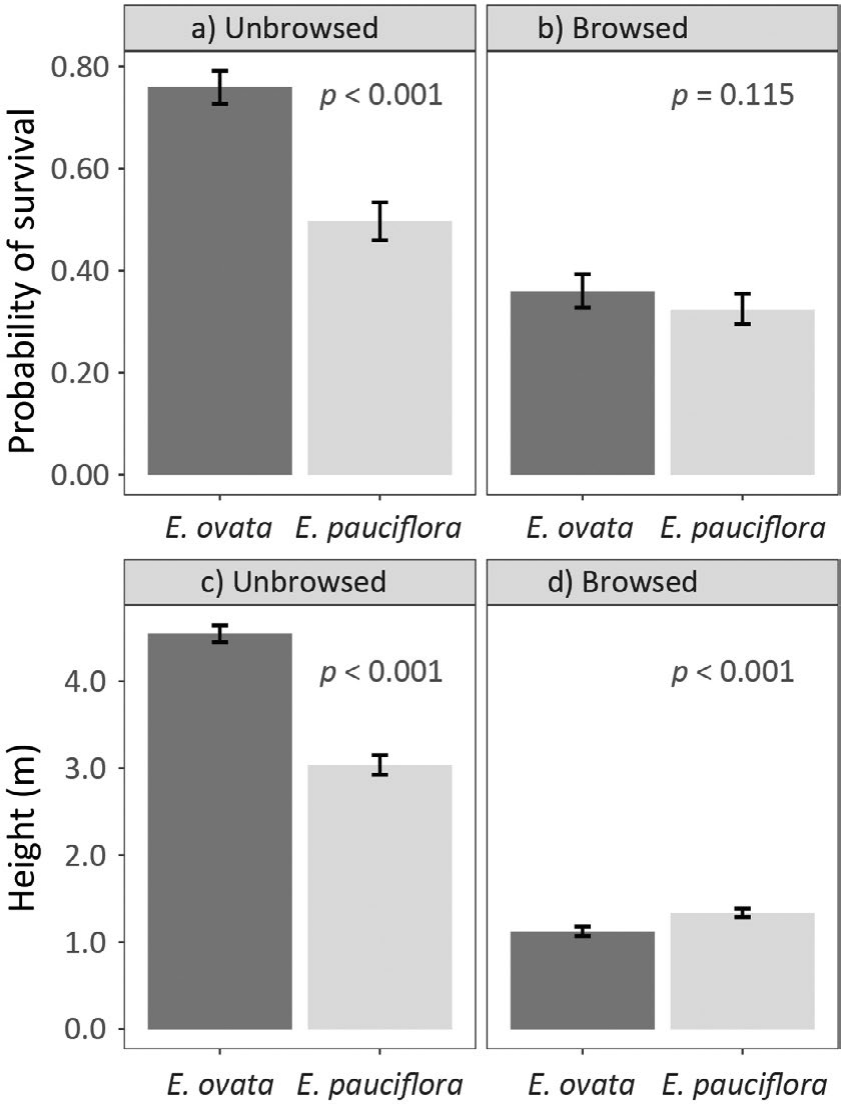
Least‐squares means for the expected probability of 8‐year‐old survival and the overall 3‐year‐old height growth estimated for mixed species field trials of *Eucalyptus ovata* and *E. pauciflora* , established in two browsing regimes—unbrowsed and browsed plantings. The 95% confidence intervals are depicted for the estimated least‐squares means, and the significance probabilities obtained from statistical *F*‐tests of the difference between species are presented for each browsing regime (see also Table S4).

In the absence of browsing, the height and survival of *E. ovata* were significantly (*p* < 0.001) greater than *E. pauciflora* (Figure 3; Tables S3 and S4). The advantage of *E. ovata* over *E. pauciflora* was removed in the presence of browsing (Figure 3; Table S4), which resulted in a significant reduction in the survival and height of both species when compared to the unbrowsed planting (Tables S3 and S5). This reduction differed between species (interaction of Species × Browsing regime, *p* < 0.001; Table 1), and was more marked in *E. ovata* (Figure 3; Table S5). The species difference in height was reversed in the presence of browsing (*p* < 0.001), but for survival, the difference was small in magnitude and not significant (Figure 3).

**TABLE 1:** Results obtained from tests of statistical significance (values of the *F*‐statistic and associated significance probabilities within parentheses) undertaken for the fixed‐effect terms (species, browsing regime, geographic area and the two‐ and three‐way interactions involving these terms) included in the definition of a mixed‐effects model fitted for data of tree survival or height growth combined across the two target species ( *Eucalyptus ovata* and *E. pauciflora* ) and browsing regimes (unbrowsed and browsed plantings).

Model term	Tree survival	Height growth
Species	67.83 (*p* < 0.001)	234.12 (*p* < 0.001)
Browsing regime	308.19 (*p* < 0.001)	3668.22 (*p* < 0.001)
Geographic area	2.81 (*p* < 0.001)	3.89 (*p* < 0.001)
Species × Browsing regime	51.35 (*p* < 0.001)	413.50 (*p* < 0.001)
Species × Geographic area	2.27 (*p* < 0.001)	3.73 (*p* < 0.001)
Browsing regime × Geographic area	0.97 (*p* = 0.496)	2.89 (*p* < 0.001)
Species × Browsing regime × Geographic area	1.27 (*p* = 0.189)	2.04 (*p* = 0.005)

*Note:* For tree survival, the results refer to the probit scale.

### 3.2 Population Level (Geographic Areas)

Across the common geographic areas sampled, the pattern of population differentiation in 8‐year tree survival differed between species (interaction of Species × Geographic area, Table 1), but the population differences in survival were not affected by browsing regime (i.e., insignificant interactions of Browsing regime × Geographic area and Species × Browsing regime × Geographic area; Table 1). In contrast, population differences in 3‐year height growth were affected by browsing regime (i.e., significant interactions of Browsing regime × Geographic area and Species × Browsing regime × Geographic area; Table 1), with the interaction significant in both species ( *E. ovata* , *p* = 0.002; *E. pauciflora* , *p* = 0.001; Table S5).

#### 3.2.1 *Eucalyptus ovata*

Survival and height differed among populations (i.e., geographic areas) of *E. ovata* in both the unbrowsed (survival, *p* = 0.030; height, *p* < 0.001) and browsed plantings (survival, *p* < 0.001; height, *p* < 0.001; not shown). Pearson correlation coefficients (with the hypothesis test on whether *r* differed significantly from zero being based on the Fisher's *z*‐transformation) between the least‐squares means of the *E. ovata* populations in the unbrowsed and browsed plantings were positive for both the expected probability of survival (*r* = 0.50, *p* = 0.016) and height growth (*r* = 0.42, *p* = 0.053).

**FIGURE 4:**
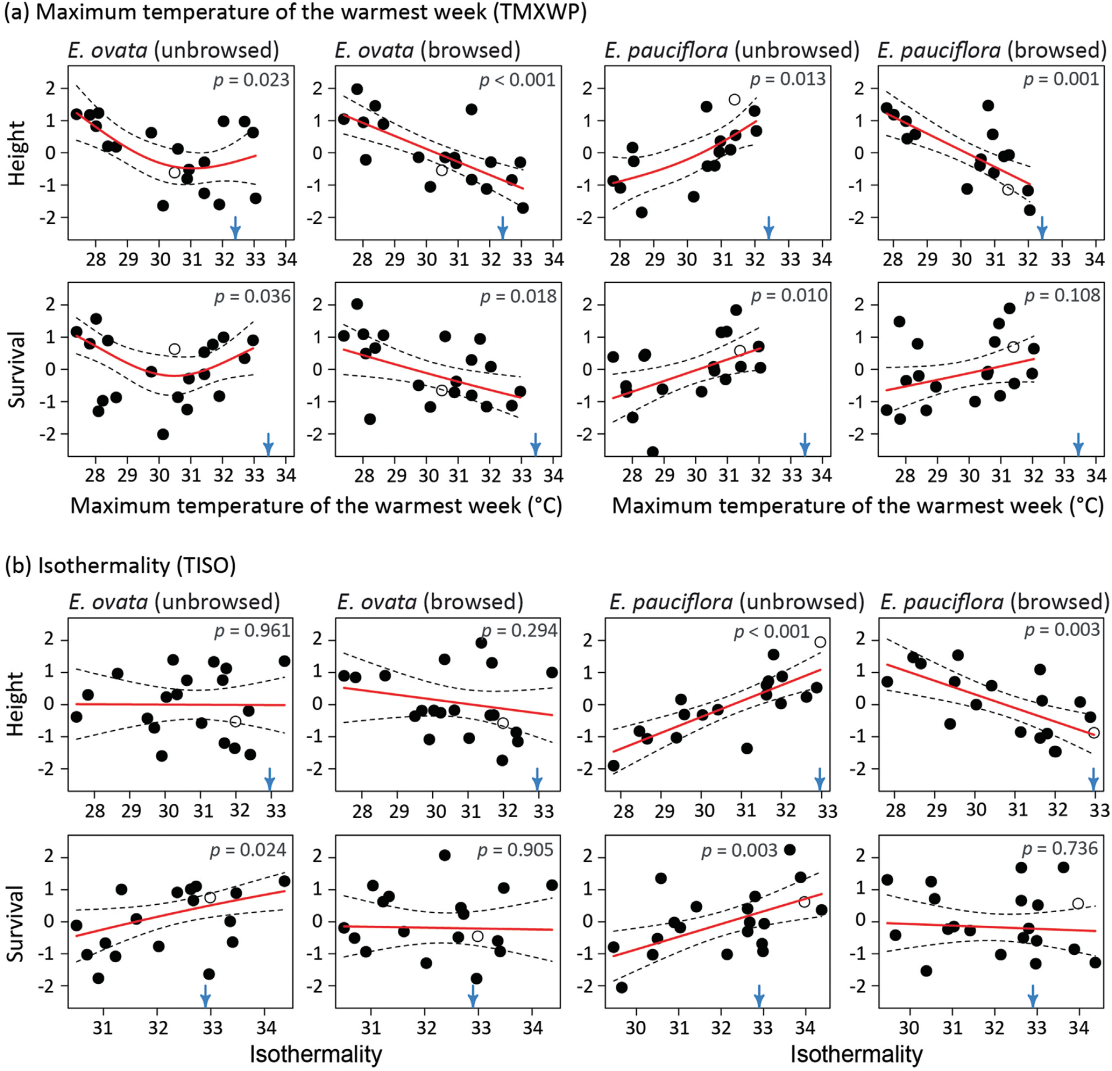
GAMs modelling the relationship of 3‐year height growth or 8‐year tree survival with climate variables for *Eucalyptus ovata* and *E. pauciflora* , established in two browsing regimes—unbrowsed and browsed plantings. In the figures, the y‐axes represent values for the height or survival response variables standardised to a unit variance, and the x‐axes pertain to home‐site climate variables for populations of each species, namely: (a) maximum temperature of the warmest week (TMXWP, °C); and (b) isothermality (TISO, % diurnal temperature range/annual temperature span). The fitted curves (red line) and corresponding 95% confidence intervals (dashed back line) were estimated on either the height response scale, or the survival probability scale (via the inverse probit link function), prior to standardisation. The symbols illustrate the standardised least‐squares means for each geographic area originally estimated from the fitted mixed‐effects models (LMMs and GLMMs for height and survival, respectively). The (local) population most proximal to the Connorville common‐garden site is shown as an open symbol, as opposed to the remaining (non‐local) populations which are illustrated with closed symbols. For each figure, the blue arrow at the bottom indicates the average of the climate variable for the 3‐year or 8‐year growing period at the common‐garden site for height and survival, respectively, and the probability (*p*‐value) at the top refers to a hypothesis test undertaken to evaluate whether a fitted model was statistically significant (note that, for survival, statistical inference refers to the probit scale; see Table 2). The results from contrasts comparing unbrowsed and browsed plantings of the same species, as well as from contrasts comparing species within the same browsing regime, are given in Table 2.

In the unbrowsed planting, the differences among *E. ovata* populations in height growth were poorly predicted by climatic variables, with a significant predictor observed for maximum temperature only (TMXWP; Tables 2 and S6). In the presence of browsing, the height differences among populations were predicted (*p* < 0.05) by maximum temperature, summer rainfall and annual rainfall (TMXWP, RWMQ and RANN; Table 2), reflecting greater height growth of populations from cooler and wetter geographic areas (Figures 4a and S2a,d). However, there were no significant differences between the pairwise models contrasting the height–climate relationship observed for the unbrowsed and browsed plantings of *E. ovata* , except for annual mean temperature (TANN, Table 2).

**TABLE 2:** Test statistics and their associated significance probabilities (*p*‐values in parentheses), obtained from hypothesis tests undertaken to evaluate whether a univariate GAM was statistically significant in the modelling of: (a) 3‐year height growth (*F* statistic shown); and (b) 8‐year tree survival (Chi‐square values shown).

Predictor	*E. ovata*			*E. pauciflora*			Species contrast	
	Unbrowsed	Browsed	Contrast	Unbrowsed	Browsed	Contrast	Unbrowsed	Browsed
(a) Height growth								
TMXWP	5.7 (*p* = 0.023)	18.2 (*p* < 0.001)	0.8 (*p* = 0.379)	5.9 (*p* = 0.013)	17.9 (*p* = 0.001)	9.3 (*p* < 0.001)	16.9 (*p* < 0.001)	3.8 (*p* = 0.047)
RWMQ	4.1 (*p* = 0.059)	14.7 (*p* = 0.001)	0.8 (*p* = 0.380)	8.8 (*p* = 0.009)	8.8 (*p* = 0.009)	7.3 (*p* = 0.001)	15.1 (*p* < 0.001)	3.9 (*p* = 0.052)
TCLQ	0.0 (*p* = 0.943)	0.9 (*p* = 0.336)	0.4 (*p* = 0.503)	10.4 (*p* = 0.005)	4.2 (*p* = 0.058)	9.4 (*p* = 0.004)	2.1 (*p* = 0.156)	0.2 (*p* = 0.652)
TISO	0.0 (*p* = 0.961)	1.2 (*p* = 0.294)	1.3 (*p* = 0.264)	22.5 (*p* < 0.001)	12.6 (*p* = 0.003)	21.6 (*p* < 0.001)	13.8 (*p* = 0.001)	0.8 (*p* = 0.387)
TANN	0.4 (*p* = 0.531)	3.1 (*p* = 0.097)	2.8 (*p* = 0.049)	14.7 (*p* = 0.001)	5.9 (*p* = 0.028)	13.3 (*p* = 0.001)	5.2 (*p* = 0.027)	0.4 (*p* = 0.541)
RANN	3.0 (*p* = 0.099)	17.0 (*p* = 0.001)	0.8 (*p* = 0.378)	4.5 (*p* = 0.062)	2.0 (*p* = 0.190)	5.2 (*p* = 0.022)	10.1 (*p* = 0.003)	3.3 (*p* = 0.032)
(b) Tree survival								
TMXWP	7.5 (*p* = 0.036)	5.6 (*p* = 0.018)	3.7 (*p* = 0.086)	6.6 (*p* = 0.010)	2.6 (*p* = 0.108)	1.7 (*p* = 0.278)	20.7 (*p* < 0.001)	24.9 (*p* < 0.001)
RWMQ	9.5 (*p* = 0.014)	7.8 (*p* = 0.005)	1.6 (*p* = 0.207)	5.6 (*p* = 0.018)	0.1 (*p* = 0.815)	1.9 (*p* = 0.169)	22.2 (*p* < 0.001)	18.4 (*p* < 0.001)
TCLQ	5.8 (*p* = 0.058)	0.0 (*p* = 0.906)	1.8 (*p* = 0.184)	11.2 (*p* = 0.001)	0.5 (*p* = 0.468)	1.0 (*p* = 0.367)	0.5 (*p* = 0.494)	0.3 (*p* = 0.731)
TISO	5.1 (*p* = 0.024)	0.0 (*p* = 0.905)	2.8 (*p* = 0.097)	8.7 (*p* = 0.003)	0.1 (*p* = 0.736)	4.1 (*p* = 0.043)	1.3 (*p* = 0.257)	1.1 (*p* = 0.293)
TANN	5.6 (*p* = 0.043)	2.0 (*p* = 0.152)	4.2 (*p* = 0.041)	6.4 (*p* = 0.011)	1.4 (*p* = 0.241)	0.1 (*p* = 0.747)	1.3 (*p* = 0.571)	4.0 (*p* = 0.044)
RANN	5.4 (*p* = 0.101)	6.2 (*p* = 0.074)	0.1 (*p* = 0.781)	5.9 (*p* = 0.016)	4.9 (*p* = 0.027)	0.0 (*p* = 0.889)	10.9 (*p* = 0.001)	13.6 (*p* = 0.001)

*Note:* For either height or survival response variable, each of six selected climate variables was used as a predictor in a univariate GAM. The results pertain to GAMs fitted separately for each species— *Eucalyptus ovata* or *E. pauciflora* —and browsing regime—unbrowsed or browsed planting—as well as to contrasts comparing browsing regimes for each species, and contrasts comparing species within each browsing regime. Full results are given for individual models in Table S6, and for the contrasts in Table S7. The predictors pertain to home‐site climate variables for the sampled populations of each species, namely: maximum temperature (TMXWP), summer rainfall (RWMQ), winter temperature (TCLQ), isothermality (TISO), annual mean temperature (TANN) and annual precipitation (RANN). For tree survival, the results refer to the probit scale.

The *E. ovata* population differences in height were reflected to some degree in differences in a fitness component, with positive correlations between the population least‐squares means estimated for the expected probability of survival and height growth in the unbrowsed (*r* = 0.54, *p* = 0.008) and browsed (*r* = 0.86, *p* < 0.001) plantings. In the browsed planting, the significant GAMs (*p* < 0.05) for height growth involving maximum temperature and summer rainfall were translated into similar GAMs for survival (TMXWP and RWMQ; Table 2; Figures 4a and S2a). By comparison, in the unbrowsed planting, significant GAMs for survival were obtained for four of the six climate predictors, despite only maximum temperature being a significant predictor of height growth (Table 2).

The GAMs for survival differed significantly between the unbrowsed and browsed plantings for annual mean temperature only (TANN; Table 2). This difference reflected relatively better survival of populations from warmer home‐sites in the unbrowsed planting, a trend that was more consistent with the similarity of the population home‐site climates to the growing period climate of the trial site but not observed in the browsed planting (TANN; Figure S2c; see also TMXWP, Figure 4a). However, for the rainfall variables, the tendency for greater height growth of populations from wetter areas in both the unbrowsed and browsed plantings was also reflected in survival (RWMQ; Figure S2a). This result counters expectations based on the similarity of the population home‐site climates to the growing period climate of the trial site. Nevertheless, the survival of the population sampled from the geographic area most proximal to the trial site (designated as the local *E. ovata* provenance—Table S2) was above average in the unbrowsed planting, but below average in the browsed planting (Figure 4). These results combined suggest that, in the browsed planting of *E. ovata* , the population 3‐year height and 8‐year survival were generally poorly predicted based on the similarity of the population home‐site climates to the growing period or contemporary climate of the common‐garden site (e.g., local population), but this was less evident in the unbrowsed planting.

#### 3.2.2 *Eucalyptus pauciflora*

In *E. pauciflora* , the populations were significantly differentiated for 3‐year height growth in the unbrowsed planting (*p* < 0.001); but population differences were not significant for height in the browsed planting (*p* = 0.565), nor for survival in both the unbrowsed (*p* = 0.213) and browsed (*p* = 0.150) plantings (not shown). Pearson correlations between the least‐squares means of the *E. pauciflora* populations in the unbrowsed and browsed plantings were not significant for height growth (*r* = −0.24, *p* = 0.290), but were positive for the expected probability of survival (*r* = 0.41, *p* = 0.060). The two studied species had independent patterns of population differentiation across the same climatic gradient as defined by the sampled geographic areas. This independence was clearly evident for height growth in the unbrowsed planting, where populations exhibited significant differences (*p* < 0.001) in both *E. ovata* and *E. pauciflora* , but the least‐squares means from the paired populations within the same geographic area were uncorrelated (*r* = −0.06, *p* = 0.781). This independence was also the case for the expected probability of survival in the unbrowsed planting (*r* = 0.03, *p* = 0.894).

In the unbrowsed *E. pauciflora* planting, all climate variables except annual precipitation (RANN) were significant (*p* < 0.05) predictors of the population differentiation of height growth (Tables 2 and S6). These GAMs were linear (except for the slight curvilinear trend in maximum temperature and annual precipitation) and reflected better height growth of populations from geographic areas with high temperature (TMXWP, TCLQ and TANN; Figures 4a and S2b,c), low rainfall (RWMQ; Figure S2a) and high isothermality (TISO; Figure 4b). The geographic area most proximal to the trial site (designated as the local *E. pauciflora* ) was amongst the hottest and driest of the geographic areas of *E. pauciflora* sampled. It had the highest 3‐year mean height growth (Figures 4 and S2) consistent with a pattern of local advantage. The population least‐squares means estimated for the expected probability of survival and height growth were positively correlated (*r* = 0.63, *p* = 0.001). Thus, as expected, the significant climate relationships observed with height in the unbrowsed planting were also reflected to varying degrees in the GAM relationships of survival (Table 2). In the unbrowsed planting, the better 3‐year height growth of the *E. pauciflora* populations from warmer and drier geographic areas was also reflected in their subsequent survival at age 8 years (e.g., Figures 4a and S2a). This finding is consistent with the expectations of local climate adaptation of populations based on their home‐site climate similarity to the growing period climate of the trial site, which was less evident in the unbrowsed planting of *E. ovata* . Indeed, for height growth, there was a significant (*p* < 0.05) pairwise contrast between the GAMs fitted for each species in the unbrowsed planting for all climate predictors except winter temperature (TCLQ; Table 2). These species differences in 3‐year height‐climate associations flowed through to the 8‐year GAMs for survival for maximum temperature (TMXWP), summer rainfall (RWMQ) and annual precipitation (RANN; Table 2; see also Figures 4a and S2a,d).

Despite the low differentiation among populations of *E. pauciflora* in the browsed planting, maximum temperature, summer rainfall, isothermality and annual mean temperature were also significant (*p* < 0.05) predictors of height growth (TMXWP, RWMQ, TISO and TANN; Table 2). However, all GAMs for the height‐climate relationship differed (*p* < 0.05) between the browsing regimes (Table 2), and the height‐climate relationships were actually reversed for most climate variables (Figures 4 and S2). Further, as opposed to the unbrowsed planting, in the browsed planting the population least‐squares means for 3‐year height growth were not significantly correlated with those estimated for the expected probability of 8‐year survival (*r* = 0.31, *p* = 0.166). The pairwise contrasts of the survival‐climate relationships in the unbrowsed and browsed plantings were not significant (*p* < 0.05), except for isothermality (TISO, Table 2; see also Figure 4b). Nevertheless, in comparison to the height‐climate relationships observed in the browsed planting of *E. pauciflora* , the directionality of most survival‐climate relationships in the unbrowsed planting changed to be either: (i) more‐or‐less neutral (e.g., TISO, Figure 4b; RWMQ, Figure S2a); or (ii) better match the height‐climate and survival‐climate relationships observed in the unbrowsed planting (e.g., TMXWP, Figure 4a; TCLQ, Figure S2b; TANN, Figure S2c; RANN, Figure S2d). Thus, the apparent reversal of the height‐climate relationships observed for *E. pauciflora* in the browsed compared to the unbrowsed planting may be a transitory effect on growth performance that did not transfer to the same trend in survival.

### 3.3 Family Variances

Separate analyses of tree survival for each combination of species and browsing regime provided ML estimates of family variances that were in close agreement with the corresponding Bayesian estimates (Table S8) and, in general, the RSPL family variances were not substantially underestimated relative to their ML counterparts used as a benchmark for comparison (for more details, see Results S1). Regardless of the estimation method, the family variances estimated for survival were reduced in the browsed compared to the unbrowsed planting (Tables 3 and S8). This decrease was most marked in *E. pauciflora* , with the family variance in the browsed planting being reduced by about 80% relative to the unbrowsed planting, compared to an approximate relative reduction of 70% for *E. ovata* (based on estimates presented in Tables 3 and S8). One‐sided likelihood‐ratio (LR) tests pursued to evaluate the statistical significance of the family variance for survival showed highly significant (*p* < 0.001) results for the unbrowsed planting in both species, but marginally significant (*p* = 0.046) and non‐significant (*p* > 0.05) results in the browsed planting in *E. ovata* and *E. pauciflora* , respectively (Table S8). As family variances were statistically significant (*p* < 0.05) in both browsing regimes for *E. ovata* , the family covariance between the two plantings was modelled for this species under the final two‐level GLMM fitted to survival data combined across species and browsing regimes (see Methods S1). This resulted in a RSPL estimate of 0.94 ± 0.35 for the family correlation, with the high estimate indicating stability in the family rankings for survival across the two browsing regimes in *E. ovata* , despite the observed erosion in family variance under the browsed compared to the unbrowsed planting.

**TABLE 3:** Family variances (±SE) estimated by residual solution pseudo‐likelihood (RSPL) for tree survival, and by restricted maximum likelihood (REML) for height growth, measured in mixed species field trials of *Eucalyptus ovata* and *E. pauciflora* established in two browsing regimes—unbrowsed and browsed plantings.

Species	Tree survival		Height growth	
	Unbrowsed	Browsed	Unbrowsed	Browsed
*E. ovata*	0.155 ± 0.049^a^	0.050 ± 0.032^a^	0.171 ± 0.047^b^ (*p* < 0.001)	0.011 ± 0.013^b^ (*p* = 0.176)
*E. pauciflora*	0.111 ± 0.039^a^	0.022 ± 0.029^a^	0.245 ± 0.058^b^ (*p* < 0.001)	0.005 ± 0.012^b^ (*p* = 0.330)

*Note:* Family variances were estimated in separate analyses for each combination of species and browsing regime. For the hierarchical structure of our data—that is, (level‐1) individual trees clustered within (level‐2) families, which were clustered within (level‐3) geographic areas—the analyses used a mixed‐effects model where geographic area was modelled as a fixed‐effect term to accommodate the highest level of the data hierarchy, whereas families within geographic areas were modelled as random effects to account for the second level of clustering. Such a modelling approach pertains effectively to a two‐level, mixed‐effects model with fixed‐effect dummy variables for level‐3 entities and random intercepts for level‐2 effects, as described by McNeish and Wentzel (2017). Tree survival was modelled under a generalized linear mixed model (GLMM) that used the probit link function, and thus the corresponding tabulated estimates of family variances refer to the probit scale.

^a^ Likelihood‐ratio tests were not pursued for testing the statistical significance of family variances estimated by RSPL, since the deviance cannot be calculated from the model under the linearisation‐based RSPL approach, which precludes the use of likelihood‐ratio tests when comparing nested GLMMs for testing (co)variance components (Hedeker and Gibbons 2006). However, for comparison with the RSPL variance estimates presented above for survival, we have also obtained maximum likelihood (ML) estimates of family variances under a three‐level probit GLMM (i.e., where geographic areas and families within geographic areas were modelled as random effects) that used an adaptive Gaussian quadrature algorithm for parameter estimation, hence allowing the statistical significance of a family variance to be assessed by a likelihood‐ratio test (Pinheiro and Chao 2006; see also Methods S1). In this context, a one‐sided likelihood‐ratio test (Self and Liang 1987) was applied to test whether a ML family variance estimate for survival was significantly greater than zero, and the corresponding significance probabilities (*p*‐values) are provided (together with the ML estimates) in Table S8.

^b^ In contrast to RSPL, REML estimation enables the statistical significance of a (co)variance component to be evaluated by a likelihood‐ratio test, and the *p*‐value from a one‐sided likelihood‐ratio test undertaken for a REML family variance estimate is presented (within parentheses) for height growth.

Akin to survival, the family variance for height growth was also substantially eroded in both species under the presence of browsing (Table 3). For the REML family variances of height in both species, one‐sided LR tests showed highly significant (*p* < 0.001) estimates in the unbrowsed planting, but not in the browsed planting (*p* > 0.05; Table 3).

## 4 Discussion

Three key findings emerge from the present study. First, uncontrolled browsing and damage by large mammals may disrupt the eco‐evolutionary dynamics of restored forests by altering the competitive advantage of tree species, populations and families, and eroding genetic‐based differences in growth and survival at all levels. Second, as part of this eco‐evolutionary impact, large mammal browsing or associated damage may distort or mask signals of local climate adaptation at the population level. This impact was most evident in *E. pauciflora* but also appeared to be emerging in the faster growing *E. ovata* . Third, the association of growth performance and fitness component measures with climate variables may be species dependent, even when similar life forms are evenly sampled from along the same climatic gradient. Such species differences were most evident in the unbrowsed planting, where there was effectively no correlation of the height growth or survival of paired *E. ovata* and *E. pauciflora* populations sampled from the same geographic area, and relationships with climate variables were often significantly different and, in some cases, nearly opposing.

The functional roles of mammals as ‘ecosystem engineers’ is well‐recognised (Lacher Jr et al. 2019; Ramsay et al. 2022), including their potential to change the competitive interactions among forest species (Cushman et al. 2020; Danell et al. 2003). Such impacts may occur at multiple levels, including seed and fruit dispersal and predation, seedling establishment and damage to established trees (Cushman et al. 2020; Danell et al. 2003; Lacher Jr et al. 2019; Quin et al. 2023). We addressed only the integrated impact on young trees (from planting to 8‐years later) in the context of the uncontrolled browsing by multiple species of large mammalian herbivores. These herbivores include feral populations of the introduced European fallow deer (likely to have been the single entity contributing most to tree damage), as well as three native marsupials—the common brushtail possum, the Tasmanian pademelon and the Bennetts wallaby (Bailey, Harrison, Davidson, et al. 2021). There are many reports of selective browsing or damage to co‐occurring or interplanted *Eucalyptus* species by native marsupials, particularly the common brushtail possum (Dungey and Potts 2002; Gordon et al. 2019; Scott et al. 2002) and introduced deer (Di Stefano 2005). The common brushtail possum is a significant ground and arboreal browser of eucalypt seedlings, saplings and mature trees in Tasmania (Calder and Kirkpatrick 2008; Dungey and Potts 2002; O'Reilly‐Wapstra et al. 2002; Scott et al. 2002). The European fallow deer browse eucalypt foliage, as well as cause damage to stems and branches (Bailey et al. 2014; Davis et al. 2016; Guy et al. 2024). This damage is distinctive, often seasonal and usually results from male deer ‘fraying’ activities, which include rubbing their heads on tree stems during antler shedding, as well as vegetation thrashing during displays, which lead to stripped bark, broken branches and stems, and ultimately tree death in severe cases (Bailey et al. 2014). Observations of eucalypt plantings in the United Kingdom suggest that deer favour two‐year‐old trees for fraying, which can see small plantings destroyed in extreme cases (Leslie and Purse 2016). For even‐aged field trials planted in Tasmania, early‐age deer damage has been reported as being most prolific on the faster growing eucalypt species (Bailey et al. 2014; Camarretta, Harrison, Bailey, et al. 2020). Such preference for the faster growing species in an even‐aged planting would potentially explain the greater reduction in growth and survival of *E. ovata* compared to the slower growing *E. pauciflora* in the present study (Figure 3), with *E. pauciflora* thus being more competitive in the presence of deer. However, these eucalypt species are from different eucalypt subgenera and differ markedly in functional traits (Prober et al. 2022) and foliar chemistry (Li et al. 1995, 1996) which could also contribute to herbivore browsing choice. Regardless of the mechanism, our study provided evidence that, at the observed period of the life cycle, *E. pauciflora* appeared more competitive with *E. ovata* when exposed to large mammalian herbivores, particularly the European fallow deer. However, we cannot dismiss the possibility that this difference will change as the trees age, recover and become less susceptible to ground‐based mammal herbivores (Skarpe and Hester 2008).

The potential of such trophic interactions for modifying community and eco‐evolutionary responses to climate change is well‐recognised (Åkesson et al. 2021; Dobor et al. 2024; Hamann et al. 2021; Leimu et al. 2012). Modelling suggests that herbivores can modify the response and speed of plant adaptation, including having a strong negative effect on the rate of adaptation to the abiotic effects of climate change (Mellard et al. 2015). While empirical studies are few, population differences in herbivory did not affect the expression of local adaptation in reciprocal transplant trials of the annual monkeyflower (Kooyers et al. 2019). In contrast, mammalian herbivory was shown to limit the response of lowland forb populations to applied warming and fertiliser treatments after their translocation to higher altitude tundra (Kaarlejärvi et al. 2013). Accordingly, the authors suggested that such herbivory may limit warm‐adapted plants from expanding their range to higher altitudes and latitudes. In our study, and for *E. pauciflora* in particular, we showed that browsing and other damage from large mammalian herbivores eroded population differences and family variance in growth performance and a fitness component, and masked signals of local climate adaptation. In *E. pauciflora* , the best populations for height growth and survival in the unbrowsed planting were from home‐sites with climates most similar to the growing period climate of the common‐garden site. This was particularly the case for home‐site temperature variables, which feature in many morphological trait‐climate relationships reported in *E. pauciflora* (Gauli et al. 2015), suggesting they are historic environmental drivers of local adaptation. In our unbrowsed planting, most height‐climate relationships were also reflected in similar climate relationships with a later‐age fitness component (8‐year survival), consistent with size‐dependent mortality over a similar growth period reported at the individual tree level in *E. pauciflora* (Costa e Silva et al. 2022) and *E. ovata* (Costa e Silva et al. 2024). However, over the period studied, the growth performance and fitness advantages of the *E. pauciflora* populations from hotter and drier home‐sites observed in the unbrowsed planting were not evident in the browsed planting. Nevertheless, the possibility that these relationships will emerge following recovery from early‐age browsing damage (Borzak et al. 2016) cannot be dismissed.

There are multiple mechanisms by which herbivores may affect the evolutionary trajectory of a target plant species, including adaptive or non‐adaptive plasticity masking genetic variation in climate adaptation, as well as selective filtering driven by herbivore susceptibility. Selective filtering has been shown in other forest tree systems, where mammalian herbivores act as agents of natural selection on plant traits which are often strongly inherited, such as phytochemistry (Bailey et al. 2007, 2004). In Australia, native marsupial herbivory has been shown to alter plant traits outside of exclosures (Cain et al. 2024), although these impacts may confound natural selection and trait plasticity. Within eucalypt species, genetic variation in susceptibility to browsing or other damage by mammalian herbivores has been shown at multiple scales, with effects on growth performance and fitness (Moore and Foley 2005; O'Reilly‐Wapstra and Cowan 2010). One of the most striking examples of population differences involves the main population of the endangered species *E. morrisbyi*, which has high susceptibility to browsing by the common brushtail possum that is associated with genetic‐based differences in physical and chemical properties of the foliage and shown to have fitness consequences (Mann et al. 2012). In the case of *E. globulus* , field trials have shown a genetic basis of variation in foliage susceptibility to uncontrolled browsing by the common brushtail possum at the population and family levels (O'Reilly‐Wapstra et al. 2002), which is genetically independent of susceptibility to pathogen and insect enemies (O'Reilly‐Wapstra et al. 2014), and linked to plant defensive chemistry (O'Reilly‐Wapstra et al. 2004).

In the present study, the observed differences in the patterns of population height growth and survival between the unbrowsed and browsed plantings most likely reflect population differences in susceptibility to mammalian browsing and other damage. There is evidence from another Tasmanian field trial that *E. pauciflora* populations differ in their susceptibility to deer damage, with the faster growing, lower altitude populations more susceptible (Bailey et al. 2014). Deer targeting faster growing, locally adapted populations from warmer home‐sites would explain the observed reversal of some height‐climate trends between the unbrowsed and browsed plantings in *E. pauciflora* . In *E. ovata* , the different height‐climate and survival‐climate relationships between the unbrowsed and browsed plantings would be consistent with mammalian herbivores targeting populations from warmer and drier as opposed to the colder and wetter geographic areas (e.g., TMXWP; Figure 4a) regardless of tree size. This targeting of the warmer and drier populations of *E. ovata* suggests that another herbivore‐tree dynamic is also at play in our system, involving a trade‐off between browsing susceptibility and climate adaptation (Agrawal 2020; Blumenthal et al. 2020). Thin leaves with high specific leaf area (SLA) affect leaf heat exchange and photosynthesis, which have been shown to decrease and increase, respectively, with population home‐site maximum temperature (TMXWP) in both *E. ovata* and *E. pauciflora* (Prober et al. 2022). However, thinner leaves with higher SLA are often less tough, less well defended and favoured by mammalian browsers (Agrawal 2020; Loney et al. 2006; Steinbauer 2001), which would result in herbivores targeting populations from hotter and drier home‐sites. Both deer fraying and foliage susceptibility to mammalian herbivory may be confounded in *E. pauciflora* and contribute to the apparent reversal of the height‐climate relationships across browsing regimes. Other mechanisms apart from differences in herbivore susceptibility may also have contributed to the observed different patterns of population differences in height growth and survival between our unbrowsed and browsed plantings, including population differences in tree architectural traits (Camarretta, Harrison, Lucieer, et al. 2020) and growth/photosynthetic responses (Brookhouse and Bi 2009; Slatyer 1977), which have been observed among populations of *E. pauciflora* . Population differences in tolerance and recovery mechanisms may also contribute (Fornoni 2011). Such mechanisms used by the evergreen eucalypts include physiological upregulation of photosynthesis (Barry and Pinkard 2013) and rapid recovery of leaf area by re‐sprouting from protected epicormic buds on the stems and branches and, in more extreme cases, from basal lignotubers (Nicolle 2006; Potts and Pederick 1999). For example, genetic‐based differences in the size of the basal lignotuber among populations of both *E. ovata* and *E. pauciflora* have been observed (Costa e Silva et al. 2021; Gauli et al. 2015; Harrison 2017), which may influence browsing recovery.

The different impacts of mammalian herbivory on the observed population differences, as well as on the height‐climate and survival‐climate associations, in *E. ovata* and *E. pauciflora* are indicative of: (i) a broader decoupling of their patterns of population differentiation in many morphological traits (Prober et al. 2022); and (ii) multiple differences in their height‐climate and survival‐climate associations in our unbrowsed planting (e.g., TMXWP, RWMQ and RANN). Our study of population differences revealed a completely different eco‐evolutionary dynamics of the two species, despite populations being sampled from across the same climatic gradient. In general, in the unbrowsed planting, early‐age population height growth in *E. ovata* was poorly associated with climate but was well associated in *E. pauciflora* (Table 2). For 8‐year survival, some climate predictors showed similar trends in *E. ovata* to *E. pauciflora* within a browsing regime (e.g., TISO; Figure 4b), but other trends were significantly different. For example, in the unbrowsed planting, populations from both the colder and wetter home‐sites and the hotter and drier home‐sites were favoured in *E. ovata* , but only those from the hotter and drier home‐sites were favoured in *E. pauciflora* (e.g., TMXWP and RWMQ; Figures 4a and S2a), which is more consistent with local climate adaptation. This dichotomy in *E. ovata* may reflect different growth strategies of wet and dry populations, and it is noteworthy that performance‐based selection on leaf traits has been reported to differ significantly in the strength and form of selection acting on individuals from wet and dry population groups (Costa e Silva et al. 2024). Populations of this species from the hotter and drier home‐sites may be favoured over the studied growth period at the common‐garden site because they are expected to be physiologically better adapted to avoid or tolerate water and heat stress (Prober et al. 2022). The populations from cooler and wetter home‐sites in *E. ovata* may also be equally favoured as their fast early growth allows the development of a larger root system, and thus better capture of resources and avoidance of water stress under competition during the establishment phase of the restoration planting (Gonçalves et al. 2013). However, it is possible that as the trees age and conditions become more stressful, selection may act against phenotypes from colder and wetter home‐sites.

## 5 Conclusions

While we have only studied a short interval in the life of two long‐lived forest tree species and a single fitness component, we provide insights into the complex nature of the eco‐evolutionary dynamics at play in even‐aged restoration plantings of native tree species at an important life‐history stage. Of key significance is our evidence that, in the presence of large mammalian herbivores, the variation in growth performance and tree survival is eroded at multiple genetic levels, the effects of local climate adaptation are masked and the competitive advantages are changed at the species, population and family levels. This finding has implications for species and provenance choices for restoration in the face of climate change, including the application of genetic rescue (Hoffmann et al. 2021) and assisted migration (Stanturf et al. 2024) or climate‐adjusted provenancing (Prober et al. 2015) as a mitigation strategies to accelerate adaptive gains for future climate resilience. There has been previous concern that mammalian browsers may prevent the objectives of such climate adaptation strategies from being achieved (reviewed for ungulates by Champagne, Raymond, et al. 2021). However, we also show that, even when occupying the same climate landscape in their natural distribution, focal tree species may exhibit different eco‐evolutionary dynamics, with marked differences in the manner that local climate adaptation affects population growth, survival and response to mammalian herbivores. Multiple mechanisms are likely to contribute to the changed eco‐evolutionary dynamics observed in the presence of large mammalian herbivores. This complexity was no doubt accentuated in our case by the multi‐species nature of the herbivore community involved, interacting with a diverse range of local and non‐local populations of a native tree species. However, such a system is likely representative of many tree restoration plantings worldwide which, in the face of global change, increasingly include translocated populations of tree species interacting with co‐evolved and alien enemies (Champagne, Royo, et al. 2021).

The extent to which the large mammalian herbivore community will continue to have a major impact on tree fitness and will shape the evolutionary trajectory of restoration plantings beyond their establishment phase is unclear, given that many of the herbivore species are ground‐based browsers, coupled with the future possibility of exotic species management (Davis et al. 2016). Indeed, as Hargreaves et al. (2020) noted, ‘biotic interactions often fail to drive local adaptation even though they strongly affect fitness, perhaps because temperate biotic environments are unpredictable at the spatiotemporal scales required for local adaptation’. Local adaptation may also manifest differently across different life stages (Wadgymar et al. 2022) and, in the present case, it is possible that, as stresses accumulate with age (Mendham et al. 2011) and the climate continues to warm (Bryant et al. 2024; Harrison 2021), signals of climate (mal)adaptation will become more prominent even under conditions similar to those of our browsed planting. Nevertheless, consistent with Champagne, Raymond, et al. (2021) and Champagne, Royo, et al. (2021), our study cautions against making provenance choices for translocation solely based on climate predictions alone, given the multiple herbivore and other biotic (Felker‐Quinn et al. 2011) interactions which may disrupt the climate relationships with growth and/or fitness attributes, and the potential differences in the geographic patterns of climate adaptation within co‐occurring tree species.

## Conflicts of Interest

The authors declare no conflicts of interest.

## Supporting information


Table S1.

## Data Availability

The data supporting the findings of this study can be requested via the University of Tasmania's Research Data Portal, https://dx.doi.org/10.25959/39t2‐ns10.
