# Supplementary material for: Mammalian Browsers Disrupt Eco‐Evolutionary Dynamics in a Forest Tree Restoration Planting
Source: Evol Appl. 2025 May 7;18(5):e70099. doi: 10.1111/eva.70099 (PMC12059210; doi:10.1111/eva.70099)
Supplement: Supplementary file 1 — Table S1. [file EVA-18-e70099-s001.pdf]

Supporting Information

Article title: Mammalian browsers disrupt eco-evolutionary dynamics in a forest tree restoration planting

Authors: [redacted]

Contents:

1. Supporting methods

Methods S1: Definition and estimation of the mixed-effects models fitted to tree survival (pages 2 to 6) and height growth (page 7).

Methods S2: Climate variables and principal components analysis (page 8).

Methods S3: Contemporary home-site climates versus growing period climate (page 9).

Methods S4: Modelling the height-climate relationship (page 10-11).

Methods S5: Modelling the survival-climate relationship (page 12).

2. Supporting results

Results S1: Comparison of family variances estimated for tree survival by different methods (page 13).

3. Supporting tables

Table S1 (page 14).

Table S2 (pages 15 to 16).

Table S3 (page 17).

Table S4 (page 18).

Table S5 (page 19).

Table S6 (page 20).

Table S7 (page 21).

Table S8 (page 22).

4. Supporting figures

Figure S1 (page 23).

Figure S2 (includes Figures S2a to S2d; page 24).

5. Supporting references (pages 25 to 27).

1. Supporting methods

Methods S1: Definition and estimation of the mixed-effects models fitted to tree survival and height growth

Generalized linear mixed model for tree survival

Model specification

The following generalized linear mixed model (GLMM) was defined for survival data (i.e. the time-averaged tree survival from planting to age 8 years) combined across the two studied field trials (i.e. data combined across species and browsing regimes):

$$g(E(\mathbf{w}\,|\,\mathbf{X},\mathbf{b}_1,\mathbf{b}_2)) = \boldsymbol{\eta} = \mathbf{X}\boldsymbol{\beta} + \mathbf{Z}_1\mathbf{b}_1 + \mathbf{Z}_2\mathbf{b}_2 \qquad \text{(S1\_1)}$$

where $\mathbf{w}$ is a n x 1 vector of observations (i.e. binary outcomes of 0 or 1, where 0 = dead and 1 = alive) on tree survival (n = total number of trees); $g(.)$ is a link function relating the conditional expectation of the response variable [i.e. $E(\mathbf{w}\,|\,\mathbf{X},\mathbf{b}_1,\mathbf{b}_2) = \boldsymbol{\mu}$, where $\boldsymbol{\mu}$ denotes the mean vector] to the linear predictor $\boldsymbol{\eta} = \mathbf{X}\boldsymbol{\beta} + \mathbf{Z}_1\mathbf{b}_1 + \mathbf{Z}_2\mathbf{b}_2$; $\boldsymbol{\beta}$ is a $m \times 1$ vector of fixed-effect parameters, including the intercept, blocks within species and browsing regime, and the effects associated with the model terms of species, browsing regime, geographic area and their two- and three-way interactions; $\mathbf{b}_1$ and $\mathbf{b}_2$ are q_1 x 1 and q_2 x 1 vectors of random effects for families within geographic areas in *E. ovata* (denoted with subscript 1) and *E. pauciflora* (denoted with subscript 2), respectively; $\mathbf{X}$ is a n x m design matrix relating the observations in $\mathbf{w}$ to the pertinent combination of fixed effects in $\boldsymbol{\beta}$; $\mathbf{Z}_1$ and $\mathbf{Z}_2$ are n x q_1 and n x q_2 design matrices relating the observations in $\mathbf{w}$ to the pertinent combination of random effects in $\mathbf{b}_1$ and $\mathbf{b}_2$, respectively. The family effects in $\mathbf{b}_1$ and $\mathbf{b}_2$ were assumed to follow a multivariate normal (MVN) distribution with zero means and independent (co)variance matrices $\mathbf{G}_1$ and $\mathbf{G}_2$, respectively, so that $\begin{pmatrix}\mathbf{b}_1\\ \mathbf{b}_2\end{pmatrix} \sim \text{MVN}\left(\begin{bmatrix}0\\0\end{bmatrix}, \begin{bmatrix}\mathbf{G}_1 & 0\\ 0 & \mathbf{G}_2\end{bmatrix}\right)$. Each of the $\mathbf{b}_1$ and $\mathbf{b}_2$ vectors was composed of two sub-vectors entailing family effects in the unbrowsed (i.e. fenced, denoted with subscript f) and browsed (i.e. unfenced, denoted with subscript u) planting regimes - i.e., $\mathbf{b}_1 = (\mathbf{b}'_{1_f}, \mathbf{b}'_{1_u})'$ and $\mathbf{b}_2 = (\mathbf{b}'_{2_f}, \mathbf{b}'_{2_u})'$ (where ' denotes the transpose operation), with their associated design matrices $\mathbf{Z}_1 = (\mathbf{Z}_{1_f}, \mathbf{Z}_{1_u})$ and $\mathbf{Z}_2 = (\mathbf{Z}_{2_f}, \mathbf{Z}_{2_u})$, respectively - so that the (co)variance matrices $\mathbf{G}_1$ and $\mathbf{G}_2$ were defined as:

$$\mathbf{G}_1 = \text{Var}\begin{pmatrix}\mathbf{b}_{1_f}\\ \mathbf{b}_{1_u}\end{pmatrix} = \begin{bmatrix}\sigma^2_{\mathrm{b}_{1_f}} & \sigma_{\mathrm{b}_1}\\ \sigma_{\mathrm{b}_1} & \sigma^2_{\mathrm{b}_{1_u}}\end{bmatrix} \otimes \mathbf{I}_{q_1} \qquad \text{(S1\_2)}$$

and

$$\mathbf{G}_2 = \mathrm{Var}\begin{pmatrix} \mathbf{b}_{2_f} \\ \mathbf{b}_{2_u} \end{pmatrix} = \begin{bmatrix} \sigma^2_{\mathrm{b}2_f} & \sigma_{\mathrm{b}_2} \\ \sigma_{\mathrm{b}_2} & \sigma^2_{\mathrm{b}2_u} \end{bmatrix} \otimes \mathbf{I}_{q_2} \qquad \text{(S1\_3)}$$

where, for a given species, the diagonal elements refer to family variances in the unbrowsed and browsed plantings, and the off-diagonal elements refer to the family covariance between the two browsing regimes; $\mathbf{I}_{q_1}$ and $\mathbf{I}_{q_2}$ are identity matrices of dimensions q_1 and q_2 (which equal the total number of families represented in *E. ovata* and *E. pauciflora*, respectively, under the full modelling of three parameters in each of the **G** matrices, as defined above); ⊗ denotes the Kronecker product operation. Thus, the complete variance-covariance matrix of the random effects accounts for heterogeneity of family variances among species and browsing regimes, besides a covariance between browsing regimes for a given family in a species. Conditional on the random family effects, each observation in $\mathbf{w}$ was assumed to be independently Bernoulli distributed, with the corresponding parameter in $\boldsymbol{\mu}$ pertaining to the conditional probability of tree survival [i.e. $P(\mathbf{w}=1\,|\,\mathbf{X},\mathbf{b}_1,\mathbf{b}_2)$]. The probit link function was used to model survival, such that:

$$P(\mathbf{w}=1\,|\,\mathbf{X},\mathbf{b}_1,\mathbf{b}_2) = g^{-1}(\boldsymbol{\eta}) = \Phi(\mathbf{X}\boldsymbol{\beta} + \mathbf{Z}_1\mathbf{b}_1 + \mathbf{Z}_2\mathbf{b}_2) \qquad \text{(S1\_4)}$$

where $g^{-1}(.)$ is the inverse of $g(.)$ and $\Phi(.)$ is the standard normal cumulative distribution function. The choice of the probit link function for modelling survival was based on the conclusions obtained by Hahn and Soyer (2005) in regard to model fit for GLMMs with multivariate binary response data.

Estimation of (co)variance parameters under the GLMM described in Equation (S1_1) relied on the residual solution pseudo-likelihood (RSPL) method (Wolfinger and O'Connell 1993; for further information, see “Frequentist parameter estimation” below). Firstly, the family variance was estimated by RSPL in separate analyses of survival for each combination of species and browsing regime, under a reduced two-level probit GLMM with blocks and geographic areas as fixed-effect parameters, and random intercepts for families within geographic areas. Then, the family variances estimated in these separate analyses were used as initial parameter values in the RSPL estimation of the two-level GLMM defined to fit the survival data combined across species and browsing regimes, as described in Equation (S1_1).

We have considered that modelling a covariance parameter in Equations (S1_2) and (S1_3) would be meaningful only if the family variances had been previously found to be statistically significant ($p < 0.05$) in both browsing regimes simultaneously within a species. This was the case for *E. ovata*, but not for *E. pauciflora* (see “Frequentist parameter estimation” below for details on the statistical testing of the family variance parameter in separate analyses of survival for each combination of species and browsing regime). Consequently, for *E. pauciflora*, the family covariance between browsing regimes was assumed to be zero - i.e., σ_{b_2} = 0 in Equation (S1_3). In addition, this was pursued to prevent a non-positive definite (co)variance matrix of the random effects in the RSPL estimation of the GLMM described in Equation (S1_1), which is more likely to occur when the level-1 sample size is not sufficiently large (as possibly in our case; see Table S1) for estimating an inherently small level-2 variance, as indicated by Schoeneberger (2016). On the other hand, searching for a positive definite solution by the RSPL method was not expected to be affected by the information available in our data in regard to the number of families (which was reasonably large for either species; Table S1) and to the prevalence of the binary outcome (as the observed proportions of tree survival were not extreme; Table S3) (Schoeneberger 2016).

Estimates of the fixed-effect parameters in $\boldsymbol{\beta}$ were obtained by generalized least-squares under the modelling of the final GLMM with five random-effect parameters (i.e. with a diagonal $\mathbf{G}_2$ matrix, as implied by $\sigma_{b_2} = 0$). The Kenward and Roger (2009) methods were applied to adjust the model-based sampling (co)variance matrix of the $\hat{\boldsymbol{\beta}}$ estimates, and to approximate the denominator degrees of freedom in (partial) F-tests used for statistical inference about the fixed-effect model terms. In particular, such an approximation of the denominator degrees of freedom for the F-statistics was considered to be adequate, as our design comprised moderately unbalanced data (Table S1) and a complex (co)variance structure (Schaalje et al. 2002). The variance of the Pearson residuals estimated from each of the analyses of survival (comprising the reduced and final GLMMs) was reasonably close to one, suggesting no model misspecification.

Frequentist parameter estimation

GLMMs for binary outcomes are nonlinear models in which likelihood functions cannot be derived in closed form. Consequently, frequentist parameter estimation in these models relies broadly on two main approaches: (a) approximation of the model (rather than the likelihood function) by linearizing its nonlinear components; or (b) approximation of the marginal likelihood function by evaluating its integral with respect to the random effects, while preserving the nonlinearity of the model (e.g. Kim et al. 2013; McNeish 2016; Pinheiro and Chao 2006).

With a complex model specification, linearization-based methods pursued under approach (a) may be advantageous in reducing the computational time used for parameter estimation (while also potentially providing easier convergence) since they do not involve integration over the distribution of random effects, as required in methods based on maximum likelihood (ML) estimation under approach (b) (Hedeker and Gibbons 2006; Kim et al. 2013; Pinheiro and Chao 2006). Such a model complexity was present in the GLMM described in Equation (S1_1) for fitting the survival data across trials, as evidenced by the complete variance-covariance matrix defined for the family random effects [i.e. Equations (S1_2) and (S1_3)], and the need for ultimately estimating five random-effect parameters (i.e. four variances and one covariance) in the modelling of the final GLMM. Thus, parameter estimation under this two-level GLMM relied on the linearization-based approach (a) by using the residual solution pseudo-likelihood (RSPL) iterative method (Wolfinger and O'Connell 1993). In addition, unlike ML estimation, the linearization-based RSPL method allows for the Kenward and Roger (2009) adjustments to be applied for improving the statistical inference about the fixed-effect parameters (McNeish 2016).

Despite the above-mentioned benefits, reported drawbacks of RSPL [or similar linearization-based methods as the penalized quasi-likelihood (PQL) proposed by Breslow and Clayton (1993)] for two-level GLMMs with binary responses entail potential downward bias particularly in level-2 variance estimates, but also a tendency for a negative impact on the (absolute) magnitude of the fixed-effect parameter estimates, and their standard errors. These problems may occur especially with: (i) small sample sizes for level-1 individuals (observations) per level-2 group; (ii) a fairly large underlying variance of the level-2 random effects (and associated intraclass correlation); and/or (iii) a proportion of an event (binary outcome prevalence) that is near zero or one (Breslow and Lin 1995; Callens and Croux 2005; Capanu et al. 2013; Diaz 2007; Kim et al. 2013; Schoeneberger 2016). Another issue with employing a linearization-based approach is that the deviance cannot be calculated from the model, as the underlying objective function for optimization is not truly an approximation to the likelihood function, hence precluding the use of likelihood-ratio tests when comparing nested GLMMs for testing (co)variance components, or the use of likelihood-based information criteria for model fit assessment and model selection (Hedeker and Gibbons 2006; Pinheiro and Chao 2006).

For ML estimation of model parameters in GLMMs, Pinheiro and Chao (2006) proposed an adaptive Gaussian quadrature (AGQ) algorithm to approximate the marginal density of the response variable by numerical integration of the joint likelihood function with respect to the random effects. The accuracy of the AGQ approximation to the marginal likelihood function of a GLMM increases with the number of quadrature points used in the numerical integration, but the processing time to obtain parameter estimates will be influenced by the complexity of the model being fitted, as well as by the sample sizes and information contained in the data (Pinheiro and Chao 2006). Therefore, for a moderately large number of cluster entities, the AGQ algorithm can be computationally-intensive (and eventually may lead to converge problems) due to the high-dimensional integrals involved when modelling several random parameters (which may be correlated), particularly in GLMMs fitted to clustered data with multiple hierarchical levels (Hedeker and Gibbons 2006; Kim et al. 2013). As noted above, such a model complexity also applies to the complete variance-covariance matrix of the random effects included in Equation (S1_1), with the estimation of the (co)variance parameters for family effects being based on a reasonably large (≥ 147) number of families per species and browsing regime, as well as a total of 5059 individuals being used for estimation of model parameters in the combined data (Table S1).

Regardless of the limitations referred to above, likelihood-ratio tests can be applied to assess the statistical significance of the ML (co)variance estimates, as the AGQ approximates the likelihood function thus enabling the deviance to be calculated (Hedeker and Gibbons 2006; Pinheiro and Chao 2006). In addition, reported analyses of binary responses by three-level GLMMs modelling only two variance parameters - i.e., random intercepts at the levels 3 and 2 of the data hierarchy - indicated nearly unbiased ML estimates of model parameters from Gaussian quadrature when compared to linearization-based methods, which notably underestimated the level-2 variance, while estimates of fixed-effects and the level-3 variance were affected to a lesser extent. These results were indicated by analyses of simulated (Capanu et al. 2013; Pinheiro and Chao 2006; Rodríguez and Goldman 2001) or empirical (Rodríguez and Goldman 2001) cross-sectional clustered data, with large numbers for the level-3 and level-2 random groups, few and sparse numbers of level-1 individuals per level-2 group, and high variance of the level-2 random effects leading to a substantial (≥ 0.38) intraclass correlation among individuals of the same level-2 group. In particular, for empirical data with these features, and under a GLMM with level-3 and level-2 random intercepts, Rodríguez and Goldman (2001) informally validated ML parameter estimates from Gaussian quadrature by finding a general agreement with estimates from Bayesian analysis, and thus used the ML approach as a benchmark against which the applied linearization-based methods were compared in their analyses of binary responses.

As mentioned in the Materials section of the article, we used a AGQ algorithm (Pinheiro and Chao 2006) in the present study for ML parameter estimation in separate analyses of survival for each combination of species and browsing regime, under a three-level probit GLMM with random intercepts for (level-3) geographic areas and (level-2) families within geographic areas, and blocks as fixed effects. The intention of these analyses was to use the ML family variance estimates for two main purposes: (i) as a benchmark for comparison with the RSPL family variances estimated under the reduced two-level probit GLMM defined separately for each combination of species and browsing regime, as described in the previous section; and (ii) to pursue a one-sided likelihood-ratio test (Self and Liang 1987) to evaluate whether a family variance parameter was significantly greater than zero. In each of these analyses, the level-3 and level-2 random effects were specified under the assumption of being normally distributed with zero means and a variance-covariance matrix defined as $\begin{bmatrix} \sigma_a^2 \mathbf{I}_k & 0 \\ 0 & \sigma_b^2 \mathbf{I}_q \end{bmatrix}$ where: σ_a^2 and σ_b^2 are the variances for geographic areas and families within geographic areas, respectively; $\mathbf{I}_k$ and $\mathbf{I}_q$ are identity matrices of dimension equal to the number of geographic areas and families, respectively, modelled for a given combination of species and browsing regime. Five quadrature

points were considered to provide sufficient accuracy in our data for integrating the likelihood function over the distribution of random effects by using the AGQ algorithm, as increasing further the number of quadrature points did not produce changes in the estimated ML variances. The procedure GLIMMIX implemented in the SAS 9.4 software (SAS 2017) was used in the frequentist data analyses.

Bayesian parameter estimation

For comparison with the ML family variance estimates, we also undertook Bayesian Markov Chain Monte Carlo (MCMC) estimation of model parameters in separate analyses of survival for each combination of species and browsing regime under a three-level probit GLMM, as defined for the ML estimation using the AGQ algorithm. The prior distributions for the probit coefficients associated with the intercept and block parameters were assumed to be normal distributions with mean 0 and a variance of 10. Such a specification has been considered by McClintock et al. (2014) as a weakly informative prior for the regression coefficients in a probit model allowing for individual heterogeneity (i.e. subject-level variation) in response probabilities.

The level-3 and level-2 random effects were specified as described above for the ML estimation. For the variance components associated with these effects, we assumed half-Cauchy prior distributions with location and scale parameters of 0 and 1, respectively; we applied these priors on the standard deviations σ_a and σ_b, rather than the variances σ_a^2 and σ_b^2, as described in Gelman (2006). The half-Cauchy distribution has a broad peak at zero, and its use has been indicated in hierarchical models when considering a weakly informative prior for the Bayesian estimation of a variance parameter, while also helping to avoid unrealistic high values in the posterior distribution of the parameter (e.g. Gelman 2006; Lemoine 2019; Röver et al. 2021; Sheng 2017). As the previous analyses using the AGQ did not suggest ML variance estimates of substantial magnitude (either for level-3 or level-2 random effects), the scale parameter of the half-Cauchy distribution was set to a value that was not too large (i.e. 1), and thus intended to provide a certain amount of prior information that attempted to constrain the posterior estimation of a variance component to lie within a reasonable range. Yet, given particularly the considerable number of families available for the estimation of σ_b in each combination of species and browsing regime (Table S1), the use of the half-Cauchy (0, 1) as a weakly informative prior may allow the information in the observed data to influence more (or dominate) the posterior distribution of the family variance parameter (Gelman 2020).

For each combination of species and browsing regime, the Bayesian analysis of survival was undertaken by specifying 100000 burn-in iterations, 1000000 MCMC iterations and thinning by 10, using the slice sampling algorithm (Neal 2003) implemented in the SAS procedure MCMC (SAS 2017) to sample from the target (posterior) distribution of a parameter. Visual inspection of trace plots, as well as non-statistically significant results ($p > 0.05$) from the Heidelberger and Welch's stationary test (1983) and the Geweke's convergence diagnostic (1992), indicated reasonable mixing and convergence of the MCMC chains for the model parameters. Point (i.e. the median) and credible interval (i.e. the 95% highest posterior density - HPD - interval) estimates were obtained from the marginal posterior distributions of the parameters. The square of the posterior median of σ_b was used as a posterior point estimate for σ_b^2, which was then compared to the ML estimate of σ_b^2 obtained from the analysis of survival in the corresponding combination of species and browsing regime.

Linear mixed model for height growth

The following linear mixed model (LMM) was defined for height growth data combined across species and browsing regimes:

$$\mathbf{y} = \mathbf{X}\boldsymbol{\beta} + \mathbf{Z}_1\mathbf{b}_1 + \mathbf{Z}_2\mathbf{b}_2 + \mathbf{e} \qquad \text{(S1\_5)}$$

where $\mathbf{y}$ is a n x 1 vector of observations on height growth; the vectors for the fixed- and random-effect model terms, and associated design matrices, in the two-level LMM described in Equation (S1_5) were defined as for the GLMM in Equation (S1_1); $\mathbf{e}$ is a n x 1 vector of random residuals of the observations. For the family random effects in $\mathbf{b}_1$ and $\mathbf{b}_2$, the distributional assumptions and the definition of the complete variance-covariance matrix were the same as described for the GLMM. It was further assumed that the random effects in these vectors were independent of the residuals in $\mathbf{e}$. The vector $\mathbf{e}$ was partitioned as $\mathbf{e} = (\mathbf{e}'_{1_f}, \mathbf{e}'_{1_u}, \mathbf{e}'_{2_f}, \mathbf{e}'_{2_u})'$, with the subscripts for species (i.e. 1 and 2) and browsing regime (i.e. *f* and *u*) being defined as before. Conditional on the family random effects, the residuals in $\mathbf{e}$ were assumed to follow a multivariate normal distribution with zero means, and to be independent across species and browsing regimes, so that their variance-covariance matrix was specified as:

$$\mathrm{Var}\begin{pmatrix} \mathbf{e}_{1_f} \\ \mathbf{e}_{1_u} \\ \mathbf{e}_{2_f} \\ \mathbf{e}_{2_u} \end{pmatrix} = \begin{bmatrix} \sigma^2_{\mathrm{e}_{1_f}}\mathbf{I}_{1_f} & 0 & 0 & 0 \\ 0 & \sigma^2_{\mathrm{e}_{1_u}}\mathbf{I}_{1_u} & 0 & 0 \\ 0 & 0 & \sigma^2_{\mathrm{e}_{2_f}}\mathbf{I}_{2_f} & 0 \\ 0 & 0 & 0 & \sigma^2_{\mathrm{e}_{2_u}}\mathbf{I}_{2_u} \end{bmatrix} \qquad \text{(S1\_6)}$$

where the diagonal elements refer to residual variances in a given combination of species and browsing regime, hence allowing to accommodate a heterogeneous residual variance structure; $\mathbf{I}_{1_f}$, $\mathbf{I}_{1_u}$, $\mathbf{I}_{2_f}$ and $\mathbf{I}_{2_u}$ are identity matrices each having a dimension that equals the number of trees in the corresponding combination of species and browsing regime.

Family and residual variance components for height were estimated by restricted maximum likelihood (REML; Patterson and Thompson 1971). Akin to the RSPL estimation for survival, the REML family variance for height was initially estimated in separate analyses for each combination of species and browsing regime with a reduced two-level LMM modelling blocks and geographic areas as fixed-effect parameters, and random intercepts for families within geographic areas. In these analyses, one-sided likelihood-ratio tests (Self and Liang 1987) indicated that our condition for modelling a covariance parameter (as mentioned above for the GLMM) could not be fulfilled in either species, as neither of them showed statistically significant ($p < 0.05$) family variances for height in both browsing regimes simultaneously. Therefore, the variance-covariance matrix of the family random effects in the REML estimation of the LMM described in Equation (S1_5) was specified as a diagonal matrix [i.e., σ_{b_1} and σ_{b_2} in Equations (S1_2) and (S1_3), respectively, were assumed to be zero]. Estimates of the fixed-effect parameters in $\boldsymbol{\beta}$ were obtained by generalized least-squares under the modelling of the final LMM with four parameters for the family random effects, and again the Kenward and Roger (2009) methods were used for statistical inference about the fixed-effect model terms based on (partial) *F*-tests.

Methods S2: Climate variables and principal components analysis

Using the contemporary climate averages (1976 - 2005), we summarised the climate variation among the studied 22 geographic areas with a principal component (PC) analysis of the correlation matrix among the standardised 19 bioclimatic variables (see Table MS2 below). In this context, we used the "prcomp" function of the *stats* package (version 4.1.2) in R and retained the first three PCs that explained 87% of the total variation in the climate data by using a broken-stick method (Borcard et al. 2011). Following Costa e Silva et al. (2018), we then identified the climatic variables that had the highest Pearson correlation (r) with each PC axis ($|r| > 0.7$), and that were also relatively independent of the variation along the other PC axes (as assessed by $|r| < 0.5$). Accordingly, three temperature variables - maximum temperature (TMXWP), winter temperature (TCLQ) and isothermality (TISO) - and one rainfall variable - summer rainfall (RWMQ)] were selected to describe the variation in contemporary climate among the geographic areas studied. These four variables were highly correlated ($|r| > 0.9$) with one of the three PCs, but poorly correlated ($|r| < 0.35$) with the others (Table MS2). These independent climate variables, as well as the more traditional variables of annual precipitation (RANN) and annual mean temperature (TANN), were the focus of our analysis of the relationship of climate with height growth or tree survival.

Table MS2 Pearson correlation coefficients of the 19 BIOCLIM variables with the first three principal components (PCs; % of total variation explained is shown in parentheses). The variables identified from this analysis as best summarising the climatic variation for each PC axis are indicated in Bold, and their correlations shown in red text.

BIOCLIM description	Units	Code	PC1 (44%)	PC2 (27%)	PC3 (16%)
Annual mean temperature	˚C	TANN	-0.47	0.87	0.1
Annual mean diurnal range	˚C	TMDR	-0.69	-0.32	0.61
Isothermality	%	**TISO**	-0.34	0.12	**0.91**
Temperature seasonality	%	TCVAR	-0.42	-0.6	0.66
Maximum temperature of the warmest period [1]	˚C	**TMXWP**	**-0.91**	0.08	-0.11
Minimum temperature of the coldest period	˚C	TMNCP	-0.03	0.93	-0.35
Annual temperature range	˚C	TSPAN	-0.73	-0.57	0.16
Temperature of the wettest quarter	˚C	TWETQ	-0.71	0.5	-0.29
Temperature of the driest quarter	˚C	TDRYQ	-0.21	0.81	0.41
Temperature of the warmest quarter	˚C	TWMQ	-0.54	0.78	0.27
Temperature of the coldest quarter [2]	˚C	**TCLQ**	-0.31	**0.95**	-0.06
Annual precipitation [3]	mm	RANN	0.9	0.24	0.33
Precipitation of the wettest period	mm	RWETP	0.89	0.15	0.09
Precipitation of the driest period	mm	RDRYP	0.34	-0.23	-0.54
Precipitation seasonality	%	RCVAR	0.35	0.08	0.11
Precipitation of the wettest quarter	mm	RWETQ	0.88	0.26	0.37
Precipitation of the driest quarter	mm	RDRYQ	0.93	0.14	0.21
Precipitation of the warmest quarter [4]	mm	**RWMQ**	**0.94**	0.01	-0.07
Precipitation of the coldest quarter	mm	RCLQ	0.79	0.3	0.46

Footnotes [1-4] - For the ease of reading, these variables are referred to in the manuscript as: [1] maximum temperature, [2] winter temperature, [3] annual precipitation and [4] summer rainfall.

Methods S3: Contemporary home-site climates versus growing period climate

To quantify the extent to which the contemporary home-site climates of the studied geographic areas of each species differed from the average of the growing period climate of the Connorville common-garden site, paired *t*-tests were pursued using the "t.test" function of the *stats* package in R.

Based on the paired *t*-tests, the growing period climate of the common-garden site was significantly ($p < 0.001$) warmer than contemporary home-site climates of the populations sampled for both species, as indicated by the annual mean temperature (TANN), maximum temperature (TMXWP) and winter temperature (TCLQ) (see Table MS3 below). These differences suggest the hypothesis that, if height growth and tree survival at the Connorville site reflect differences in climate adaptation among the populations sampled, then populations from the warmer geographic areas would be favoured. The growing period isothermality (TISO) of the common-garden site was also significantly ($p < 0.001$) lower than the contemporary average of TISO for the population home-sites, reflecting a relatively greater increase in the annual than daily temperature range during the growing period. On average, the annual precipitation (RANN) for the growing period in the common-garden site was 32 mm greater than that experienced at the population home-sites (Table MS3), but the first three years of the growing period (i.e. until height was measured) were drier, and below the contemporary rainfall experienced at the majority of population home-sites (e.g. RWMQ and RANN; Figures S2a and S2d).

Table MS3 Results of the paired *t*-tests comparing the growing period climate (2014 - 2021) of the Connorville common-garden site to the home-site contemporary climate (1976 - 2005) for populations of *E. ovata and E. pauciflora*. Climate variable codes and units follow Table MS2 in Methods S2. For the variables identified from principal component (PC) analysis as best summarising the climatic variation (indicated in Bold; Methods S2), the specific PC highly correlated ($|r| > 0.9$) with the climate variable is indicated (last column of the table). For each climate variable, the table shows the mean difference between the growing period climate of the common-garden site and the contemporary climate of the population home-sites, its lower and upper 95% confidence limits (CLs), the value of the *t*-statistic, the degrees of freedom (df) and the probability associated with the null hypothesis of a mean difference being equal to zero (*p*-value).

Climate variable	Mean difference	Lower CL	Upper CL	*t*	df	*p*-value	
E. ovata							
TANN	0.46	0.44	0.49	34.69	21	< 0.001	
**TISO**	-0.49	-0.63	-0.35	-7.33	21	< 0.001	PC3
**TMXWP**	1.25	0.99	1.50	10.12	21	< 0.001	PC1
**TCLQ**	0.27	0.24	0.30	19.35	21	< 0.001	PC2
RANN	31.95	15.32	48.58	3.99	21	0.001	
**RWMQ**	10.50	0.18	20.82	2.12	21	0.046	PC1
E. pauciflora							
TANN	0.46	0.43	0.49	34.36	21	< 0.001	
**TISO**	-0.53	-0.64	-0.41	-9.26	21	< 0.001	PC3
**TMXWP**	1.29	1.04	1.53	11.00	21	< 0.001	PC1
**TCLQ**	0.27	0.25	0.30	20.31	21	< 0.001	PC2
RANN	32.16	14.16	50.15	3.72	21	0.001	
**RWMQ**	11.78	0.71	22.85	2.21	21	0.038	PC1

Methods S4: Modelling the height-climate relationship

The relationship of height growth with each focal climate variable was modelled separately for each species and browsing regime, using a Gaussian generalised additive model (GAM) with an identity link function, fitted with the "gam" function of the *mgcv* package in R (Wood 2017), under the following specification:

$$y = \beta_0 + s(x_i) + \varepsilon \qquad \text{(S4\_1)}$$

where the response variable y is the least-squares mean estimated for the height growth of a geographic area; β_0 is the intercept; $s(.)$ is a penalised thin-plate smoother with three basis functions, where the final number of basis functions was selected using generalised cross-validation; x_i is the ith climate variable retained from the PC analysis (see above); and ε is the residual term. The use of three basis functions was selected to be large enough so that the model can reasonable approximate the trend, but small enough to avoid overfitting. Although initial exploration found no detectable patterning when plotting the standardised Pearson residuals against the estimated standard errors of the least-squares means, the use of the latter as a response in Equation (S4_1) nonetheless violates the assumption that a dependent variable is measured without error, potentially affecting the estimates of regression coefficients and the statistical inference about their effects. Therefore, to increase the accuracy and precision of the parameter estimates in the height-climate models, the response observations were weighted by using the inverse of the squared values of their estimated standard errors, and normalized by dividing the weights by their mean following Wood (2017), so as not to alter the overall magnitude of the likelihood scaling.

Model assumptions of normality and homogeneity of the residuals were graphically explored using plots produced by the "gam.check" function of the *mgcv* package, which indicated potential outlier observations that may influence the statistical interpretation of the model. Therefore, to ensure that the height-climate relationships were not affected by observations with high influence or leverage, separate models were fitted using Equation (S4_1) but fixing the unpenalized smoother parameter to zero (i.e. fitting a GAM model with fx = TRUE) and setting the number of basis functions to four, thus effectively fitting a linear model. The leverage and influence of an observation were then estimated using (respectively): (i) the diagonal of the hat matrix from the "influence.gam" function of the *mgcv* package; and (ii) an approximation of the Cook's distance. Influential points were identified as those observations that exceeded the threshold defined by $4/(n - k - 1)$, where n is the sample size and k is the number of predictor variables, and further graphically explored to assess their impact on the model's overall fit of the data. After removing observations with substantial influence when compared to other observations, models were subsequently refitted and model assumptions re-examined. In all cases, these final models adequately met the assumptions of residual normality and homoscedasticity. To evaluate the approximate significance of the smoother term for each final model, a Wald F-test was pursued using the "summary.gam" function of the *mgcv* package in R. This approach uses the Bayesian covariance matrix of the smoother values to test the null hypothesis that the smooth function is equal to zero (Wood 2012). Fitted curves and associated 95% confidence intervals (CIs) from the final models were obtained by using predictions of the response variable for "new climate data", representing a vector of 200 even-spaced observations within the bound of the minimum and maximum values of a given climate predictor. The fitted curves and their 95% CIs where standardised to a unit variance for plotting, to aid comparisons across species and browsing regimes.

To test if the smoother of the relationship of height with each climate variable differed between species and browsing regime, an ordered-factor smooth interaction GAM was pursued using the following model:

$$y = \beta_0 + X_{\beta 1} + X_{\beta 2} + s(x_i) + s\left(x_i, \text{by} = X_{\beta 2}\right) + \varepsilon \qquad \text{(S4\_2)}$$

where y is defined as described above; $X_{\beta 1}$ is a cofactor with 22 levels representing each geographical area; $X_{\beta 2}$ is an ordered factor with four levels comprising unique combinations of species and browsing regime; $s(.)$ is a thin-plate smoother with three basis functions, where the final number of basis functions was optimised using generalised cross-validation; x_i is the ith climate variable retained from the PC analysis (see above); $s(x_i$, by = $X_{\beta 2})$ is an ordered factor thin-plate smooth interaction with three basis functions, where the optimal number of basis functions was determined by generalised cross-validation; and ε is the residual term. To account for measurement error in the response variable, the observations were weighted as described above. Fitting a GAM with an ordered factor thin-plate smooth interaction invokes a different model structure, whereby the "gam" function constructs "difference smoothers" that are effectively the pairwise mean difference between the reference level and each level of the ordered factor. In this case, three models were fitted to construct four contrasts of interest, namely comparing the smooth relationship between: (i) the browsing regimes for *E. ovata* (assigning *E. ovata* in the unbrowsed planting as the reference level for the ordered factor-smooth - Model 1); (ii) the browsing regimes for *E. pauciflora* (assigning *E. pauciflora* in the unbrowsed planting as the reference level for the ordered factor-smooth - Model 2); (iii) the two species in the unbrowsed planting (Model 1); and (iv) the two species in the browsed planting (assigning *E. ovata* in the browsed planting as the reference level for the ordered factor-smooth - Model 3). Final models were fitted using the combined species dataset excluding species-specific observations with substantial influence when compared to other observations, and graphical evaluations revealed no violations of assumptions of residual normality and homoscedasticity in these models (see above). To evaluate the approximate statistical significance of these contrasts, a Wald F-test was pursued using the "summary.gam" function of the *mgcv* package in R.

Methods S5: Modelling the survival-climate relationship

The relationship of survival with each focal climate variable was also evaluated separately for each species and browsing regime, with the values of the response variable consisting of the estimated least-squares means of the expected probability of survival for each geographic area, obtained from the GLMM (see Methods S1 above). In this context, by lying in a continuous scale within the interval (0, 1), the response variable was assumed to follow a standard beta distribution, which can be parameterized as a function of a location parameter μ (i.e. mean response) and a scaling parameter ϕ (i.e. "precision" or the inverse of dispersion) (Smithson and Verkuilen 2006). We fitted ϕ as a nuisance parameter (e.g. Ferrari and Cribari-Neto 2004), and the probit link function was used for relating the linear predictor to the conditional mean (μ). The linear predictor was defined with the same model terms as previously used for the height-climate relationship (except for an explicit residual term), and the response variable was weighted as previously described. This analytical approach yielded similar results in terms of relationship patterns and statistical inference when the survival-climate relationships were alternatively modelled with the response variable comprising binomial counts defined by the number of alive trees out of the total number of trees measured for each geographic area, assuming a binomial distribution for the response variable (data not shown).

Estimates of the GAM parameters were obtained by maximum likelihood estimation. The final fitted models excluded observations that were detected to have substantial leverage and influence based on the diagnostics and graphic examination described above. In these models, plots of Pearson residuals versus fitted values did not shown a systematic pattern in residual variation (i.e. they showed an even spread of the residuals, randomly scattered above and below zero), indicating that our modelling of ϕ as a nuisance parameter appeared to be adequate, hence not suggesting the need for an explicit modelling of ϕ as a function of the focal predictor variables (e.g. as in Costa e Silva et al. 2018; Smithson and Verkuilen 2006). In addition, the estimated variance of the Pearson residuals was close to one in these models, suggesting no model misspecification.

Statistical significance of the survival-climate relationships on the probit scale was approximated by undertaking a Wald Chi-square test, using the "summary.gam" function of the *mgcv* package in R. Plots of fitted curves and associated 95% CIs based on the final models were obtained on the probability scale (i.e. via the inverse probit-link function) following the procedure described above, and standardised to a unit variance to aid comparisons across species and browsing regimes. To test if the smoother of the survival-climate relationship for each climate variable differed between species and browsing regime, an ordered-factor smooth interaction GAM was pursued similarly to what was previously described for Equation (S4_2). Subsequently, the approximate statistical significance of each of the four contrasts of interest was assessed using a Wald Chi-square test that was pursued using the "summary.gam" function of the *mgcv* package in R.

2. Supporting results

Results S1: Comparison of family variances estimated for tree survival by different methods

Separate analyses of tree survival for each combination of species and browsing regime under a three-level probit GLMM provided maximum likelihood (ML) estimates of family variances that were generally in agreement with the corresponding parameter values obtained from Bayesian estimation (i.e. the relative difference between the two methods did not exceed 10%, being 5.6% on average; Table S8). In this context, the proximity of the ML and Bayesian estimates of family variances may reflect the reasonably large number of families available for estimation (Table S1) and the use of a weakly informative prior for the family variance parameter in the Bayesian analysis (Methods S1). Yet, such results suggested that, for survival, it was plausible to use these ML estimates as a benchmark for comparison with the family variances estimated by residual solution pseudo-likelihood (RSPL) under a two-level probit GLMM defined separately for each combination of species and browsing regime, as well as for assessing whether a family variance parameter was significantly greater than zero via a one-sided likelihood-ratio test (see Methods S1 for more details).

For survival in each combination of species and browsing regime, the RSPL estimates of family variances were lower than the corresponding ML estimates, with the relative difference between the two methods being 6.5% on average (albeit it exceeded slightly 10% for the case of *E. ovata* in the unbrowsed planting; Tables 3 and S8). Therefore, in general, these results did not indicate a substantial underestimation in RSPL family variances, which possibly reflects: (i) underlying variances of the family effects that were not large in magnitude (as suggested by intraclass correlations based on ML estimates ranging from 0.022 to 0.18; see footnotes of Table S8); and (ii) prevalences of the binary outcome that were not extreme (as provided by the observed proportions of survival; Table S3) (see Methods S1 for further information and references). Nevertheless, the underestimation in RSPL family variances may express a putative downward bias due to a limited number of individuals per family (i.e. 8 trees, on average; Table S1) used in the variance estimation.

3. Supporting tables

TABLE S1 Sample sizes (with ranges provided within parentheses for average numbers) used in the establishment of the mixed species field trials of *E. ovata* and *E. pauciflora*, planted under two browsing regimes - unbrowsed and browsed plantings. The average numbers were rounded to two decimal places.

	E. ovata		*E. pauciflora*	
	Unbrowsed	Browsed	Unbrowsed	Browsed
Total No. of trees	1270	1255	1261	1273
Total No. of families	157	147	155	150
Average No. of trees per geographic area	57.73 (40 to 80)	57.05 (40 to 80)	57.32 (40 to 96)	57.86 (40 to 96)
Average No. of families per geographic area	7.14 (5 to 10)	6.68 (4 to 10)	7.05 (4 to 12)	6.82 (4 to 12)
Average No. of trees per family	8.09 (5 to 10)	8.54 (5 to 14)	8.14 (7 to 12)	8.49 (5 to 16)

TABLE S2 Location and home-site climate details for the populations of *E. ovata* and *E. pauciflora*, paired-sampled from 22 geographic areas in Tasmania. The table provides the population name, the geographic area pair, latitude, longitude, elevation, as well as contemporary (1976 - 2005) estimates of the home-site annual mean temperature (TANN) and annual precipitation (RANN). The populations indicated in bold are the geographically closest population of each species to the Connorville common-garden site, and were designated as the "local" population in the text of the article, and in Figures 4 and S2. The common-garden site is at 188 m elevation above sea level (asl).

Population	Pair	Latitude (°South)	Longitude (°East)	Elevation (m asl)	TANN (ºC)	RANN (mm)
E. ovata						
Avoca	1	-41.816	147.754	206	11.6	563
Bignells Bothwell	2	-42.403	147.104	478	9.6	570
Brushy Lagoon	3	-41.391	146.731	290	10.7	1018
**Connorville Dam**	4	-41.886	147.135	268	10.1	669
Curringa	5	-42.568	146.772	111	11.7	621
Coals Marsh	6	-41.757	148.110	581	10.3	868
Ellesmere	7	-42.408	147.291	451	10.1	551
Woods Lake	9	-42.078	147.028	749	6.8	832
Lake Leake	10	-42.010	147.795	614	9.0	800
Nunamara	11	-41.369	147.271	431	10.5	1092
Osterley	12	-42.336	146.744	395	10.2	687
Ross lowlands	13	-42.023	147.593	333	9.9	636
South Arm	14	-42.996	147.483	10	12.7	607
Epping Forest	16	-41.763	147.306	199	11.6	582
Andover	17	-42.313	147.485	429	10.4	546
Tooms Lake	18	-42.213	147.784	471	9.2	738
Tunbridge	19	-42.137	147.402	213	11.3	456
Tyne River	20	-41.470	147.830	297	10.2	1015
Uralla	21	-42.551	146.856	156	11.4	534
Mt Cameron	22	-40.954	147.817	50	13.1	823
West Oatlands	23	-42.269	147.298	437	9.6	567
Fosterville	24	-41.958	147.431	191	11.3	520
E. pauciflora						
Avoca	1	-41.709	147.834	236	11.7	612
Bignells Bothwell	2	-42.401	147.095	483	9.6	570
Brushy Lagoon	3	-41.407	146.746	278	10.7	1018
**Cressy**	4	-41.719	147.105	159	11.8	626
Curringa	5	-42.570	146.772	97	11.7	621
Dukes Marshes	6	-41.722	148.128	498	10.0	958
Ellesmere	7	-42.401	147.298	420	10.1	551
Interlaken	9	-42.146	147.141	819	7.8	697
Lake Leake	10	-42.022	147.820	608	9.0	800
Nunamara	11	-41.373	147.322	409	10.9	1028
Osterley	12	-42.354	146.741	341	10.2	687
Ross	13	-42.003	147.538	229	11.3	509
South Arm	14	-43.034	147.422	16	12.7	629
Symmons Plains	16	-41.659	147.249	165	11.9	605
Tin Dish Rivulet	17	-42.307	147.436	422	10.2	546
Tooms Lake	18	-42.221	147.793	486	9.2	738

Tunbridge	19	-42.125	147.359	229	11.3	475
Tyne River	20	-41.472	147.817	297	10.8	931
Uralla	21	-42.546	146.858	196	11.4	534
Waterhouse	22	-40.911	147.662	16	13.5	717
Oatlands	23	-42.301	147.384	403	10.2	541
Fosterville	24	-41.931	147.424	185	9.9	627

TABLE S3 Proportions of tree survival and overall means for height growth observed in mixed species field trials of *E. ovata* and *E. pauciflora* established in two browsing regimes - unbrowsed and browsed plantings. Standard deviations are also provided within parentheses for height growth.

Species	Tree survival		Height growth (m)	
	Unbrowsed	Browsed	Unbrowsed	Browsed
E. ovata	0.744	0.375	4.558<br>(1.431)	1.181<br>(0.810)
E. pauciflora	0.500	0.344	3.120<br>(1.262)	1.387<br>(0.691)

TABLE S4 Results obtained from tests of statistical significance (values of the *F*-statistic, and associated significance probabilities within parentheses) undertaken for the fixed-effect terms (species, geographic area, and the interaction involving these terms) included in the definition of a mixed-effects model fitted to data of tree survival or height growth combined across the two target species (*E. ovata* and *E. pauciflora*) within each of the studied browsing regimes (unbrowsed or browsed planting). For tree survival, the results refer to the probit scale.

Model term	Tree survival		Height growth	
	Unbrowsed	Browsed	Unbrowsed	Browsed
Species	104.29<br>($p < 0.001$)	2.51<br>($p = 0.115$)	388.10<br>($p < 0.001$)	34.81<br>($p < 0.001$)
Geographic area	1.58<br>($p = 0.054$)	2.52<br>($p < 0.001$)	2.78<br>($p < 0.001$)	6.09<br>($p < 0.001$)
Species x Geographic area	1.56<br>($p = 0.060$)	2.19<br>($p = 0.002$)	2.87<br>($p < 0.001$)	2.89<br>($p < 0.001$)

In all the analyses, the mixed-effects model used a (2 x 2) variance-covariance matrix for the family random effects that was defined with a diagonal form (i.e. with family variances for each species as diagonal elements and a zero covariance between species as off-diagonal elements, since the two species did not have families in common). In the analyses of height growth, a diagonal form was also specified in the (2 x 2) variance-covariance matrix used for modelling the residual effects (i.e. heterogeneous residual variances estimated separately for each species).

TABLE S5 Results obtained from tests of statistical significance (values of the *F*-statistic, and associated significance probabilities within parentheses) undertaken for the fixed-effect terms (browsing regime, geographic area, and the interaction involving these terms) included in the definition of a mixed-effects model fitted to data of tree survival or height growth combined across the two studied browsing regimes (unbrowsed and browsed plantings) within each of the target species (*E. ovata* or *E. pauciflora*). For tree survival, the results refer to the probit scale.

Model term	Tree survival		Height growth	
	E. ovata	*E. pauciflora*	*E. ovata*	*E. pauciflora*
Browsing regime	322.77 ($p < 0.001$)	51.24 ($p < 0.001$)	3649.46 ($p < 0.001$)	733.49 ($p < 0.001$)
Geographic area	2.94 ($p < 0.001$)	1.83 ($p = 0.016$)	5.85 ($p < 0.001$)	2.06 ($p = 0.006$)
Browsing regime x Geographic area	1.51 ($p = 0.087$)	0.79 ($p = 0.733$)	2.31 ($p = 0.002$)	2.56 ($p = 0.001$)

We modelled the covariance between browsing regimes in the mixed-effects model when the family variance estimates were previously found to be statistically significant at the 5% significance level in both browsing regimes simultaneously, a condition that was intended to avoid the occurrence of a non-positive definite variance-covariance matrix for the family random effects, while also providing a more meaningful family covariance parameter between browsing regimes. This was the case of tree survival in *E. ovata* (Table S8), and thus the (2 x 2) variance-covariance matrix for families within geographic areas was parameterized with variances for each browsing regime as diagonal elements and a covariance between browsing regimes as off-diagonal elements. Otherwise, for the remaining three analyses (all of which involved a statistically non-significant family variance estimate in the browsed planting; Tables 3 and S8), the (2 x 2) variance-covariance matrix for families within geographic areas was defined with a diagonal form (i.e. assuming a zero covariance between browsing regimes). In the analyses of height growth, a diagonal form was also specified in the (2 x 2) variance-covariance matrix used for modelling the residual effects (i.e. heterogeneous residual variances estimated separately for each browsing regime).

TABLE S6 Results of the GAMs fitted to: (a) 3-year height growth; and (b) 8-year tree survival by species and browsing regime. For either height or survival response variable, each of six selected climate variables was used as a predictor in a univariate GAM. The table shows the number of populations included in the model (n pop), the effective degrees of freedom for the model (eDF) based on the optimised number of basis functions, the values of *F* and Chi-square (χ^2) statistics and their associated significance probabilities (*p*-values: $p \leq 0.10$ = .; $p \leq 0.05$ = *; $p \leq 0.01$ = **; $p \leq 0.001$ = ***), and the percentage of the observed deviance explained by the model (D^2). The climate variable codes are detailed in Methods S2. For tree survival, the results refer to the probit scale.

(a) Height growth

Variable	n pop	eDF	*F*	*p*-value		D^2	n pop	eDF	*F*	*p*-value		D^2
	E. ovata (unbrowsed)						*E. pauciflora* (unbrowsed)					
TMXWP	19	2	5.7	0.023	*	39.9	16	1	5.9	0.013	*	46.0
RWMQ	19	1	4.1	0.059	.	19.4	19	1	8.8	0.009	**	34.2
TCLQ	18	1	0.0	0.943		0.0	18	1	10.4	0.005	**	39.4
TISO	20	1	0.0	0.961		0.0	18	1	22.5	< 0.001	***	58.5
TANN	20	1	0.4	0.531		8.9	18	1	14.7	0.001	***	47.9
RANN	19	1	3.0	0.099	.	15.2	19	2	4.5	0.062	.	30.2
	E. ovata (browsed)						*E. pauciflora* (browsed)					
TMXWP	19	1	18.5	< 0.001	***	52.1	16	1	17.9	0.001	***	56.1
RWMQ	19	1	14.7	0.001	***	54.9	19	1	8.8	0.009	**	34.0
TCLQ	18	2	0.9	0.336		16.5	18	1	4.2	0.058	.	20.6
TISO	20	1	1.2	0.294		6.1	18	1	12.6	0.003	**	44.0
TANN	20	1	3.1	0.097	.	14.6	18	1	5.9	0.028	*	26.8
RANN	19	1	17.0	0.001	***	50.1	19	2	2.0	0.190		21.9

(b) Tree survival

Variable	n pop	eDF	χ^2	*p*-value		D^2	n pop	eDF	χ^2	*p*-value		D^2
	E. ovata (unbrowsed)						*E. pauciflora* (unbrowsed)					
TMXWP	20	2	7.5	0.036	*	31.6	19	1	6.6	0.010	**	28.2
RWMQ	18	2	9.5	0.014	*	41.0	19	1	5.6	0.018	*	25.2
TCLQ	17	1	5.8	0.058	.	26.1	18	1	11.2	0.001	***	41.6
TISO	18	1	5.1	0.024	*	24.5	20	1	8.7	0.003	**	32.9
TANN	18	2	5.6	0.043	*	32.1	17	1	6.4	0.011	*	30.2
RANN	19	1	5.4	0.101	.	24.0	19	1	5.9	0.016	*	25.9
	E. ovata (browsed)						*E. pauciflora* (browsed)					
TMXWP	20	1	5.6	0.018	*	24.1	19	1	2.6	0.108		13.4
RWMQ	18	1	7.8	0.005	**	32.6	19	1	0.1	0.815		0.3
TCLQ	17	1	0.0	0.906		0.1	18	1	0.5	0.468		3.2
TISO	18	1	0.0	0.905		0.1	20	1	0.1	0.736		0.6
TANN	18	1	2.0	0.152		12.1	17	1	1.4	0.241		8.5
RANN	19	1	6.2	0.074	.	25.6	19	1	4.9	0.027	*	23.3

TABLE S7 Pairwise contrasts of the GAMs fitted to: (a) 3-year height growth; and (b) 8-year tree survival. For either height or survival response variable, each of six selected climate variables was used as a predictor in a univariate GAM. The table shows the contrast being tested (i.e. comparing browsing regimes for each species, or comparing species within each browsing regime) for a given climate predictor, the number of samples included in the two models being compared (n), the effective degrees of freedom for the model (eDF) based on the optimised number of basis functions, the values of F and Chi-square (χ^2) statistics and their associated significance probabilities (p-values: $p \leq 0.10$ = .; $p \leq 0.05$ = *; $p \leq 0.01$ = **; $p \leq 0.001$ = ***). The climate variable codes are detailed in Methods S2. For tree survival, the results refer to the probit scale.

Contrast	Height growth					Tree survival				
	n	eDF	F	p-value		n	eDF	χ^2	p-value	
TMXWP										
E. ovata unbrowsed vs browsed	35	1	0.8	0.379		39	2	3.7	0.086	.
E. pauciflora unbrowsed vs browsed	35	2	9.3	< 0.001	***	39	2	1.7	0.278	
E. ovata unbrowsed vs *E. pauciflora* unbrowsed	35	1	16.9	< 0.001	***	39	2	20.7	< 0.001	***
E. ovata browsed vs *E. pauciflora* browsed	35	2	3.8	0.047	*	39	2	24.9	< 0.001	***
RWMQ										
E. ovata unbrowsed vs browsed	38	1	0.8	0.380		37	1	1.6	0.207	
E. pauciflora unbrowsed vs browsed	38	2	7.3	0.001	***	37	1	1.9	0.169	
E. ovata unbrowsed vs *E. pauciflora* unbrowsed	38	1	15.1	< 0.001	***	37	2	22.2	< 0.001	***
E. ovata browsed vs *E. pauciflora* browsed	38	2	3.9	0.052	.	37	2	18.4	< 0.001	***
TCLQ										
E. ovata unbrowsed vs browsed	36	1	0.4	0.503		35	1	1.8	0.184	
E. pauciflora unbrowsed vs browsed	36	1	9.4	0.004	**	35	1	1.0	0.367	
E. ovata unbrowsed vs *E. pauciflora* unbrowsed	36	1	2.1	0.156		35	1	0.5	0.494	
E. ovata browsed vs *E. pauciflora* browsed	36	1	0.2	0.652		35	1	0.3	0.731	
TISO										
E. ovata unbrowsed vs browsed	38	1	1.3	0.264		38	1	2.8	0.097	.
E. pauciflora unbrowsed vs browsed	38	1	21.6	< 0.001	***	38	1	4.1	0.043	*
E. ovata unbrowsed vs *E. pauciflora* unbrowsed	38	1	13.8	0.001	***	38	1	1.3	0.257	
E. ovata browsed vs *E. pauciflora* browsed	38	1	0.8	0.387		38	1	1.1	0.293	
TANN										
E. ovata unbrowsed vs browsed	38	1	2.8	0.049	*	35	1	4.2	0.041	*
E. pauciflora unbrowsed vs browsed	38	1	13.3	0.001	***	35	1	0.1	0.747	
E. ovata unbrowsed vs *E. pauciflora* unbrowsed	38	1	5.2	0.027	*	35	1	1.3	0.571	
E. ovata browsed vs *E. pauciflora* browsed	38	1	0.4	0.541		35	1	4.0	0.044	*
RANN										
E. ovata unbrowsed vs browsed	38	1	0.8	0.378		38	1	0.1	0.781	
E. pauciflora unbrowsed vs browsed	38	2	5.2	0.022	*	38	1	0.0	0.889	
E. ovata unbrowsed vs *E. pauciflora* unbrowsed	38	1	10.1	0.003	**	38	1	10.9	0.001	***
E. ovata browsed vs *E. pauciflora* browsed	38	2	3.3	0.032	*	38	1	13.6	0.001	***

TABLE S8 Maximum likelihood (ML) and Bayesian estimates of family variances for tree survival, measured in mixed species field trials of *E. ovata* and *E. pauciflora* established in two browsing regimes - unbrowsed and browsed plantings.

Species	ML estimates		Bayesian estimates	
	unbrowsed	browsed	unbrowsed	browsed
E. ovata	0.179 ± 0.059 ($p < 0.001$)	0.050 ± 0.035 ($p = 0.046$)	0.190 (0.436; HPD: 0.295, 0.577)	0.047 (0.217; HPD: 0.027, 0.361)
E. pauciflora	0.121 ± 0.044 ($p < 0.001$)	0.023 ± 0.031 ($p = 0.210$)	0.122 (0.349; HPD: 0.224, 0.474)	0.021 (0.145; HPD: 0.0004, 0.280)

Family variances were estimated in separate analyses for each combination of species and browsing regime under a three-level generalized linear mixed model (GLMM), where (level-3) geographic areas and (level-2) families within geographic areas were modelled as random effects. The GLMM used the probit link function for modelling tree survival, and thus the tabulated estimates of family variances refer to the probit scale.

The ML family variance estimates (± standard errors) were obtained from the adaptive Gaussian quadrature algorithm proposed by Pinheiro and Chao (2006); a one-sided likelihood-ratio test (Self and Liang 1987) was pursued to assess whether a ML family variance estimate was significantly greater than zero (the p-values within parentheses pertain to the significance probabilities).

For the random effects, the Bayesian estimation used half-Cauchy prior distributions on the family standard deviations rather family variances (as described in Gelman 2006), and thus the values provided within parentheses refer to estimates of the median and the 95% highest posterior density (HPD) credible interval, obtained from the marginal posterior distribution of the family standard deviation parameter. The square of the posterior median of the family standard deviation was used as a posterior point estimate for the family variance, and this estimate is provided in the table for comparison with the ML family variance for a given combination of species and browsing regime.

Further methodological details on the ML and Bayesian estimation methods of the model parameters can be found in Methods S1.

Estimation of intraclass correlation coefficients for tree survival:

Using the ML estimates of variance components for geographic areas and families within geographic areas, the dependency among observations due to the multilevel structure in the survival data was measured by the intraclass correlation coefficient (ICC). The following two alternative measures were calculated to define the ICC at the family level for tree survival:

$$\mathrm{ICC}_1 = \frac{\hat{\sigma}_b^2}{\hat{\sigma}_a^2 + \hat{\sigma}_b^2 + 1}$$

and

$$\mathrm{ICC}_2 = \frac{\hat{\sigma}_a^2 + \hat{\sigma}_b^2}{\hat{\sigma}_a^2 + \hat{\sigma}_b^2 + 1}$$

where $\hat{\sigma}_a^2$ and $\hat{\sigma}_b^2$ are the ML estimates of variances for geographic areas and families within geographic areas, respectively; the variance of 1 used in the denominator of the equations described above corresponds to the inherent linked-scale, distribution-specific variance for a GLMM using the probit link function (e.g. Nakagawa and Schielzeth 2010). Either of these ICC coefficients can be calculated to measure the dependency among observations under a 3-level mixed-effects model, with the ICC_1 evaluating the proportion of the total variance that is explained at the family level, and the ICC_2 evaluating the expected correlation between two randomly chosen individuals within the same family (Hox et al. 2017). A given ICC was calculated after controlling for the effects of the experimental blocks, and thus refers to a *conditional* measure (i.e. conditional on the fixed effects of the blocks; Rabe-Hesketh and Skrondal 2012).

The estimated values of the ICC_1 were: for *E. ovata*, 0.147 and 0.044 in the unbrowsed and browsed plantings, respectively; for *E. pauciflora*, 0.107 and 0.022, in the unbrowsed and browsed plantings, respectively.

The estimated values of the ICC_2 were: for *E. ovata*, 0.180 and 0.120 in the unbrowsed and browsed plantings, respectively; for *E. pauciflora*, 0.113 and 0.031 in the unbrowsed and browsed plantings, respectively.

4. Supporting figures

POSITION

**ROW**	**1**	**2**	**3**	**4**	**5**	**6**	**7**	**8**	**9**	**10**	**11**	**12**	**13**	**14**	**15**	**16**
**1**	PAU	OVA	PAU	OVA	PAU	OVA	PAU	OVA	PAU	OVA	PAU	OVA	PAU	OVA	PAU	OVA
**2**	OVA	PAU	OVA	PAU	OVA	PAU	OVA	PAU	OVA	PAU	OVA	PAU	OVA	PAU	OVA	PAU
**3**	PAU	OVA	PAU	OVA	PAU	OVA	PAU	OVA	PAU	OVA	PAU	OVA	PAU	OVA	PAU	OVA
**4**	OVA	PAU	OVA	PAU	OVA	PAU	OVA	PAU	OVA	PAU	OVA	PAU	OVA	PAU	OVA	PAU
**5**	PAU	OVA	PAU	OVA	PAU	OVA	PAU	OVA	PAU	OVA	PAU	OVA	PAU	OVA	PAU	OVA
**6**	OVA	PAU	OVA	PAU	OVA	PAU	OVA	PAU	OVA	PAU	OVA	PAU	OVA	PAU	OVA	PAU
**7**	PAU	OVA	PAU	OVA	PAU	OVA	PAU	OVA	PAU	OVA	PAU	OVA	PAU	OVA	PAU	OVA
**8**	OVA	PAU	OVA	PAU	OVA	PAU	OVA	PAU	OVA	PAU	OVA	PAU	OVA	PAU	OVA	PAU
**9**	PAU	OVA	PAU	OVA	PAU	OVA	PAU	OVA	PAU	OVA	PAU	OVA	PAU	OVA	PAU	OVA
**10**	OVA	PAU	OVA	PAU	OVA	PAU	OVA	PAU	OVA	PAU	OVA	PAU	OVA	PAU	OVA	PAU
**11**	PAU	OVA	PAU	OVA	PAU	OVA	PAU	OVA	PAU	OVA	PAU	OVA	PAU	OVA	PAU	OVA
**12**	OVA	PAU	OVA	PAU	OVA	PAU	OVA	PAU	OVA	PAU	OVA	PAU	OVA	PAU	OVA	PAU
**13**	PAU	OVA	PAU	OVA	PAU	OVA	PAU	OVA	PAU	OVA	PAU	OVA	PAU	OVA	PAU	OVA
**14**	OVA	PAU	OVA	PAU	OVA	PAU	OVA	PAU	OVA	PAU	OVA	PAU	OVA	PAU	OVA	PAU
**15**	PAU	OVA	PAU	OVA	PAU	OVA	PAU	OVA	PAU	OVA	PAU	OVA	PAU	OVA	PAU	OVA
**16**	OVA	PAU	OVA	PAU	OVA	PAU	OVA	PAU	OVA	PAU	OVA	PAU	OVA	PAU	OVA	PAU
**17**	PAU	OVA	PAU	OVA	PAU	OVA	PAU	OVA	PAU	OVA	PAU	OVA	PAU	OVA	PAU	OVA
**18**	OVA	PAU	OVA	PAU	OVA	PAU	OVA	PAU	OVA	PAU	OVA	PAU	OVA	PAU	OVA	PAU
**19**	PAU	OVA	PAU	OVA	PAU	OVA	PAU	OVA	PAU	OVA	PAU	OVA	PAU	OVA	PAU	OVA
**20**	OVA	PAU	OVA	PAU	OVA	PAU	OVA	PAU	OVA	PAU	OVA	PAU	OVA	PAU	OVA	PAU

FIGURE S1 An example of the alternate planting of trees of *Eucalyptus ovata* (OVA, white cells) and *E. pauciflora* (PAU, grey cells) within blocks of the field trials. There were eight blocks in both the unbrowsed (fenced) and browsed (unfenced) plantings. Families from the 22 populations of each species (147 to 157 in total per species) were generally represented once per block with the family position randomised independently in each block. There were 160 planting positions per species within each block and the few additional positions in excess of the available families were planted with fillers chosen from families of the same species.

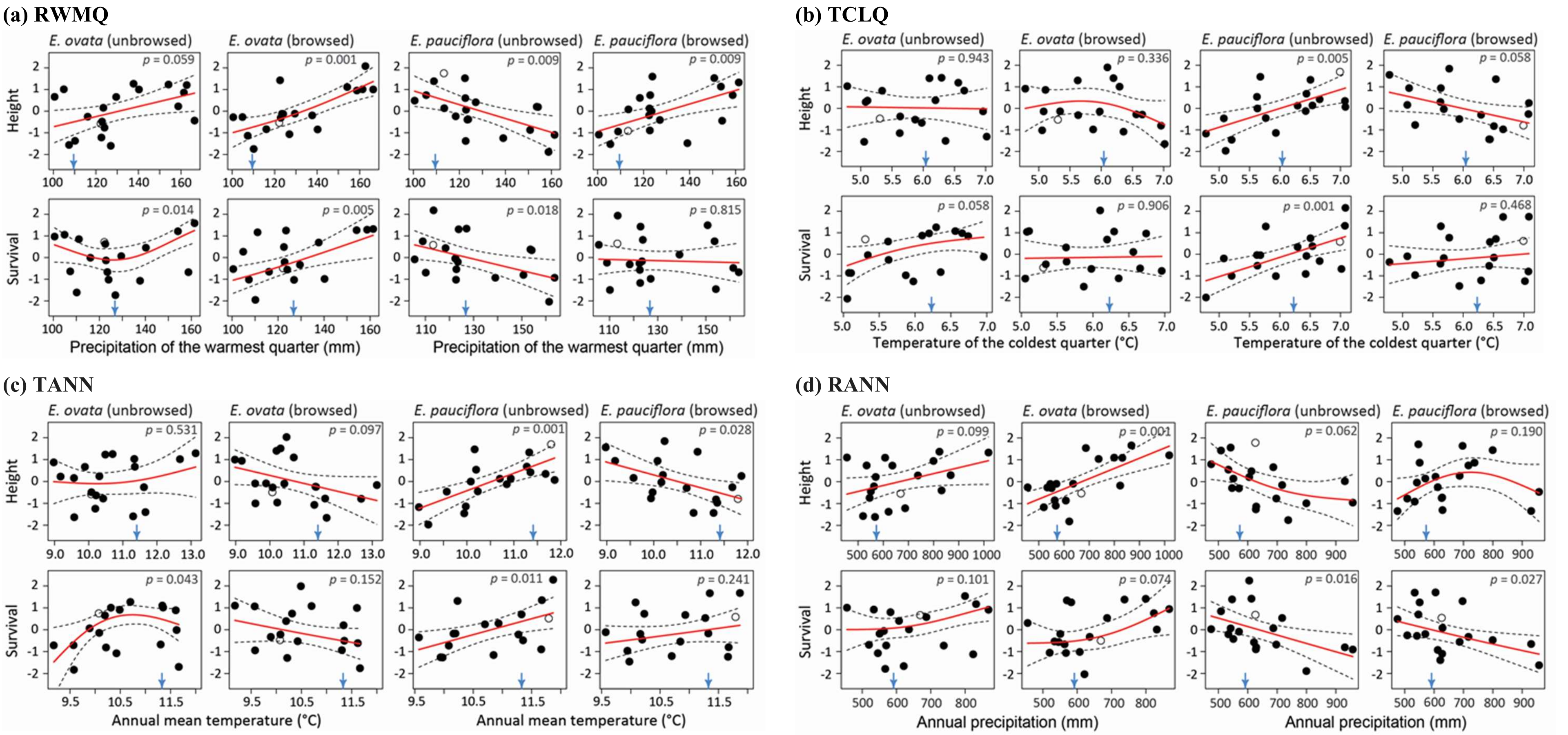

FIGURE S2 GAMs modelling the relationship of 3-year height growth or 8-year tree survival with climate variables for *E. ovata* and *E. pauciflora*, established in two browsing regimes - unbrowsed and browsed plantings. In the figures, the y-axes represent values for the height or survival response variables standardised to a unit variance, and the x-axes pertain to home-site climate variables for populations of each species, namely: (a) summer rainfall (RWMQ, mm); (b) winter temperature (TCLQ, °C); (c) annual mean temperature (TANN, °C); and (d) annual precipitation (RANN, mm). The fitted curves (red line) and corresponding 95% confidence intervals (dashed back line) were estimated on either the height response scale, or the survival probability scale (via the inverse probit-link function), prior to standardisation. The symbols illustrate the standardised least-squares means for each geographic area originally estimated from the fitted mixed-effects models (LMMs and GLMMs for height and survival, respectively). The (local) population most proximal to the Connorville common-garden site is shown as an open symbol, as opposed to the remaining (non-local) populations which are illustrated with closed symbols. For each figure, the blue arrow at the bottom indicates the average of the climate variable for the 3-year or 8-year growing period at the common-garden site for height and survival, respectively, and the probability (*p*-value) at the top refers to a hypothesis test undertaken to evaluate whether a fitted model was statistically significant (see Table 2 in the article; note that, for survival, statistical inference refers to the probit scale).

5. Supporting references

Borcard, D., Gillet, F., Legendre, P., 2011. *Numerical Ecology with R*. New York, NY: Springer.

Breslow, N.E., Clayton, D.G., 1993. Approximate inference in generalized linear mixed models. Journal of the American Statistical Association 88, 9-25.

Breslow, N.E., Lin, X., 1995. Bias correction in generalised linear mixed models with a single component of dispersion. Biometrika 82, 81-91.

Callens, M., Croux, C., 2005. Performance of likelihood-based estimation methods for multilevel binary regression models. Journal of Statistical Computation and Simulation 75, 1003-1017.

Capanu, M., Gönen, M., Begg, C.B., 2013. An assessment of estimation methods for generalized linear mixed models with binary outcomes. Statistics in Medicine 32, 4550-4566.

Costa e Silva, J., Harrison, P.A., Wiltshire, R., Potts, B.M., 2018. Evidence that divergent selection shapes a developmental cline in a forest tree species complex. Annals of Botany 122, 181-194.

Diaz, R.E., 2007. Comparison of PQL and Laplace 6 estimates of hierarchical linear models when comparing groups of small incident rates in cluster randomised trials. Computational Statistics and Data Analysis 51, 2871-2888.

Ferrari, S., Cribari-Neto, F., 2004. Beta regression for modelling rates and proportions. Journal of Applied Statistics 31, 799-815.

Gelman, A., 2006. Prior distributions for variance parameters in hierarchical models. Bayesian Analysis 1, 515-534.

Gelman, A., 2020. Prior choice recommendations, https://github.com/stan-dev/stan/wiki/Prior-Choice-Recommendations , accessed in December 2023.

Geweke, J., 1992. Evaluating the accuracy of sampling-based approaches to calculating posterior moments. In *Bayesian Statistics*, Vol. 4, edited by J.M. Bernardo, J.O. Berger, A.P. Dawid, and A.F.M. Smith, pp. 169-193. Oxford: Clarendon Press.

Hahn, E.D., Soyer, R., 2005. Probit and logit models: Differences in the multivariate realm. George Washington University. Working paper, http://home.gwu.edu/~soyer/mv1h.pdf, accessed 16 November 2020.

Heidelberger, P., Welch, P.D., 1983. Simulation run length control in the presence of an initial transient. Operations Research 31, 1109-1144.

Hedeker, D., Gibbons, R.D., 2006. *Longitudinal Data Analysis*. Hoboken, NJ: John Wiley & Sons.

Hox, J.J., Moerbeek, M., van de Schoot, R., 2017. *Multilevel Analysis: Techniques and Applications*. London: Routledge.

Kenward, M.G., Roger, J.H., 2009. An improved approximation to the precision of fixed effects from restricted maximum likelihood. Computational Statistics and Data Analysis 53, 2583-2595.

Kim, Y., Choi, Y.K., Emery, S., 2013. Logistic regression with multiple random effects: a simulation study of estimation methods and statistical packages. The American Statistician 67, 171-182.

Lemoine, N.P., 2019. Moving beyond noninformative priors: why and how to choose weakly informative priors in Bayesian analyses. Oikos 128, 912-928.

McClintock, B.T., Bailey, L.L., Dreher, B.P., Link, W.A., 2014. Probit models for capture-recapture data subject to imperfect detection, individual heterogeneity and misidentification. The Annals of Applied Statistics, 8, 2461-2484.

McNeish, D., 2016. Estimation methods for mixed logistic models with few clusters. Multivariate Behavioral Research 51, 790-804.

Nakagawa, S., Schielzeth, H., 2010. Repeatability for Gaussian and non-Gaussian data: a practical guide for biologists. Biological Reviews 85, 935-956.

Neal, R.M., 2003. Slice sampling. Annals of Statistics 31, 705-757.

Patterson, H.D., Thompson, R., 1971. Recovery of inter-block information when block sizes are unequal. Biometrika 58, 545-554.

Pinheiro, J.C., Chao, E.C., 2006. Efficient Laplacian and adaptive Gaussian quadrature algorithms for multilevel generalized linear mixed models. Journal of Computational and Graphical Statistics 15, 58-81.

Rabe-Hesketh, S., Skrondal, A., 2012. *Multilevel and Longitudinal Modeling Using Stata (3rd Edition)*. Stata Press, College Station, Texas: StataCorp LLC.

Rodriguez, G., Goldman, N., 2001. Improved estimation procedures for multilevel models with binary response: a case-study. Journal of the Royal Statistical Society, Series A (Statistics in Society) 164, 339-355.

Röver, C., Bender, R., Dias, S., Schmid, C.H., Schmidli, H., Sturtz, S., Weber, S., Friede, T., 2021. On weakly informative prior distributions for the heterogeneity parameter in Bayesian random-effects meta-analysis. Research Synthesis Methods 12, 448-474.

SAS Institute Inc., 2017. *SAS/STAT® 14.3 User's Guide*. Cary, NC: SAS Institute Inc.

Schaalje, G.B., McBride, J.B., Fellingham, G.W., 2002. Adequacy of approximations to distributions of test statistics in complex mixed linear models. Journal of Agricultural, Biological, and Environmental Statistics 7, 512-524.

Schoeneberger, J.A., 2016. The impact of sample size and other factors when estimating multilevel logistic models. The Journal of Experimental Education 84, 373-397.

Self, S.G., Liang, K.-Y., 1987. Asymptotic properties of maximum likelihood estimators and likelihood ratio tests under nonstandard conditions. Journal of the American Statistical Association 82, 605-610.

Sheng, Y., 2017. Investigating a weakly informative prior for item scale hyperparameters in hierarchical 3PNO IRT Models. Frontiers in Psychology 8, 123.

Smithson, M., Verkuilen, J., 2006. A better lemon squeezer? Maximum-likelihood regression with beta-distributed dependent variables. Psychological Methods 11, 54-71.

Wolfinger, R., O'Connell, M., 1993. Generalized linear mixed models a pseudo likelihood approach. Journal of Statistical Computation and Simulation 48, 233-243.

Wood, S.N., 2012. On p-values for smooth components of an extended generalized additive model. Biometrika 100, 221-228.

Wood, S.N., 2017. Generalized Additive Models Generalized Additive Models: *An Introduction with R (2nd edition)*. New York: Chapman and Hall/CRC.
